# A Kaposi’s sarcoma-associated herpes virus-encoded microRNA contributes to dilated cardiomyopathy

**DOI:** 10.1038/s41392-023-01434-3

**Published:** 2023-06-09

**Authors:** Yanru Zhao, Huaping Li, Hengzhi Du, Zhongwei Yin, Mengying He, Jiahui Fan, Xiang Nie, Yang Sun, Huiying Hou, Beibei Dai, Xudong Zhang, Yuanyuan Cai, Kunying Jin, Nan Ding, Zheng Wen, Jiang Chang, Chen Chen, Dao Wen Wang

**Affiliations:** 1 Division of Cardiology, Department of Internal Medicine, Tongji Hospital, Tongji Medical College, Huazhong University of Science and Technology, 430030 Wuhan, China; 2 Hubei Key Laboratory of Genetics and Molecular Mechanisms of Cardiological Disorders, 430030 Wuhan, China; 3 Division of Cardiology, Department of Internal Medicine, Hubei Provincial Renmin Hospital, 430030 Wuhan, China; 4 Division of Cardiology, Department of Internal Medicine, The First People’s Hospital of Anqing, 246004 Anqing, China; 5 Department of Epidemiology and Biostatistics, Key Laboratory for Environment and Health, School of Public Health, Tongji Medical College, Huazhong University of Science and Technology, 430030 Wuhan, China

**Keywords:** Cardiology, Molecular medicine, Inflammation

## Abstract

Dilated cardiomyopathy (DCM) is the leading cause of heart transplantation. By microRNA (miRNA) array, a Kaposi’s sarcoma-associated herpes virus (KSHV)-encoded miRNA, kshv-miR-K12-1-5p, was detected in patients with DCM. The KSHV DNA load and kshv-miR-K12-1-5p level in plasma from 696 patients with DCM were measured and these patients were followed-up. Increased KSHV seropositivity and quantitative titers were found in the patients with DCM compared with the non-DCM group (22.0% versus 9.1%, *p* < 0.05; 168 versus 14 copies/mL plasma, *p* < 0.05). The risk of the individual end point of death from cardiovascular causes or heart transplantation was increased among DCM patients with the KSHV DNA seropositivity during follow-up (adjusted hazard ratio 1.38, 95% confidence interval 1.01–1.90; *p* < 0.05). In heart tissues, the KSHV DNA load was also increased in the heart from patients with DCM in comparison with healthy donors (1016 versus 29 copies/10^5^ cells, *p* < 0.05). The KSHV and kshv-miR-K12-1-5p in DCM hearts were detected using immunofluorescence and fluorescence staining in situ hybridization. KSHV itself was exclusively detectable in CD31-positive endothelium, while kshv-miR-K12-1-5p could be detected in both endothelium and cardiomyocytes. Moreover, kshv-miR-K12-1-5p released by KSHV-infected cardiac endothelium could disrupt the type I interferon signaling pathway in cardiomyocytes. Two models of kshv-miR-K12-1-5p overexpression (agomiR and recombinant adeno-associated virus) were used to explore the roles of KSHV-encoded miRNA in vivo. The kshv-miR-K12-1-5p aggravated known cardiotropic viruses-induced cardiac dysfunction and inflammatory infiltration. In conclusion, KSHV infection was a risk factor for DCM, providing developmental insights of DCM involving virus and its miRNA (https://clinicaltrials.gov. Unique identifier: NCT03461107).

## Introduction

As a final common consequence of cardiovascular diseases, heart failure (HF) is a global epidemic affecting more than 26 million individuals worldwide.^1^ Dilated cardiomyopathy (DCM) is currently the second most common cause of HF.^2^ The prognosis of patients with DCM has been improved with the implementation of therapeutic strategies and earlier diagnosis in the last decades, but the treatment is still challenging.^3^ The underlying etiologies of DCM are varied and include genetic mutations, infectious agents (particularly viruses), toxins like alcohol, chemotherapeutic agents, autoimmune and systemic disorders.^4^ Biopsy-proven viral myocarditis has been reported in 9–16% of adult patients with DCM, and up to 30% of these patients with viral myocarditis might progress to DCM.^5^ In a cohort of 172 patients with DCM, genomes of viruses including parvovirus B19 (PB19), enteroviruses, human herpesvirus 6 (HHV6), and adenoviruses have been detected in 8.1–36.6% of endomyocardial biopsies.^6^ The viral persistence and activated immune responses may lead to the cardiomyocyte degeneration, cardiac remodeling and thereby the development of DCM.^7^ Presence of viral genome, such as PB19, adenovirus and Epstein–Barr virus (EBV), in endomyocardial biopsies, was an independent risk factor for graft loss in pediatric cardiac transplant recipients.^8^ Meanwhile, in addition to cardiomyocytes, viruses found in human myocardium samples may infect other types of cells.^9^ It has been reported that PB19 could infect cardiac endothelium and speculatively cause endothelial damage and thereby cardiac ischemia and dysfunction.^10^ In vitro, HHV6 could infect human endothelial cells and increase their ability to secrete chemokines.^11^ However, the theory of a viral pathogenic basis for myocarditis-induced cardiomyopathy was mainly supported by the determination of viral RNA, DNA, protein or neutralizing antibody in patients with DCM.^7,12^ The underlying association between viral infection and the development of DCM requires further study.

Type I interferons (IFNs) are produced by cells in response to microbial products and consist of seven classes, IFNα, IFNβ, IFNω, IFNκ, IFNε, IFNδ and IFNτ, in which IFNα and IFNβ are the most extensively studied.^13^ Type I IFNs act on most cell types and are important innate immune mediators against virus infection. Binding of type I IFNs to their heterodimeric receptor interferon-α/β receptor (IFNAR) activates the IFN-stimulated gene factor 3/signal transducer and activator of transcription 1 homodimers and induces downstream interferon-sensitive response element (ISRE)-driven genes encoding antiviral proteins such as 2′–5′-oligoadenylate synthetase (OAS), MX dynamin like GTPase (MX), interferon-stimulated genes (ISGs) and interferon regulatory factor (IRF).^14^ The downstream genes of type I IFNs provide powerful and diverse defense mechanisms against viruses. Indeed, OAS proteins activate endoribonuclease RNase L to cleave viral RNAs, while MX proteins prevent viral replication at early time points by mediating vesicle trafficking to trap essential viral components.^15^ Mice with impaired type I IFN signaling pathway suffered severe myocarditis after coxsackievirus B3 (CVB3) infection and long-term viral persistence.^16^ IFNβ-1β treatment led to effective virus clearance or substantial decrease of the viral load and improved functional performance of the patients with chronic viral cardiomyopathy.^17^ Viral genomic material persisting in patients’ heart contributed to chronic inflammation and development of DCM.^18^ Thus, disruption of type I IFN signaling pathway may have set in motion the process of DCM.

microRNAs (miRNAs) are a class of well-characterized small non-coding RNAs that regulate gene expressions via binding to the target gene mRNA and mediating its degradation or translation inhibition. Dysregulated miRNAs have been reported to be important in cardiovascular diseases.^19^ Apart from the host-originated miRNAs, miRNAs encoded by the genomes of invasive viruses were found to regulate biological and pathological processes in the infected host.^20^ A human cytomegalovirus-derived miRNA was shown to target IRF1 and independently correlated with an increased risk of essential hypertension.^21^ However, limited study has reported the association between viral miRNAs and DCM to our best knowledge. Interestingly, recently we found that five miRNAs encoded by viruses were upregulated in the heart tissues of patients with DCM compared to healthy donors, while 30 viral miRNAs were increased in the plasma of patients with DCM.^22^ Remarkably, 3 of the upregulated cardiac miRNAs and 7 of the upregulated plasma miRNAs were encoded by Kaposi’s sarcoma-associated herpes virus (KSHV).

KSHV, also known as HHV8, is the causative agent of Kaposi’s sarcoma (KS), primary effusion lymphoma, and multicentric Castleman’s disease.^23^ Recent studies have also reported KSHV infection was associated with a significant elevation in the risk of osteosarcoma and idiopathic pulmonary fibrosis.^24,25^ KSHV encodes 12 pre-microRNAs, which are processed into 25 mature miRNAs to regulate viral and its host cellular gene expressions.^26^ Increased KSHV-encoded miRNAs have been found in patients with sepsis, while they acted as agonists of Toll-like receptor 8 (TLR8) and contributed to cytokine dysregulation.^27^ Moreover, we found that only one of these KSHV-encoded miRNAs, kshv-miR-K12-1-5p, was elevated both in the heart and plasma of DCM patients with HF,^22^ which was selected for further studied. Therefore, the aims of the present study were to determine the potential association among kshv-miR-K12-1-5p, KSHV, and DCM progression involving the anti-viral effects of type I IFN signaling pathway.

## Results

### KSHV-encoded miRNA and KSHV infection were increased in patients with DCM

**Fig. 1:**
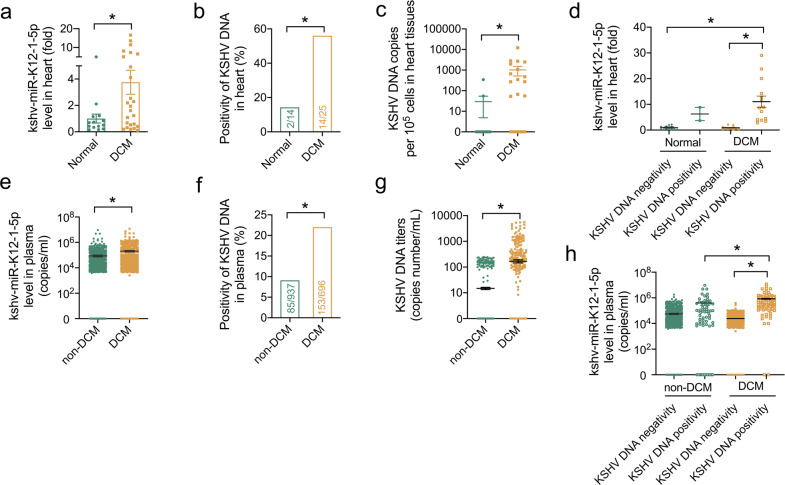
KSHV-encoded miRNA and KSHV infection were increased in patients with DCM. **a** Relative levels of kshv-miR-K12-1-5p in the heart from normal (healthy donors, *n* = 14) and DCM patients (*n* = 25). **b** The positivity of KSHV DNA in the heart from normal (healthy donors, *n* = 14) and DCM patients (*n* = 25). **c** The KSHV DNA copies in the heart from normal (healthy donors, *n* = 14) and DCM patients (*n* = 25). **d** Relative levels of kshv-miR-K12-1-5p in the heart from normal with KSHV DNA negativity (*n* = 12), normal with KSHV DNA positivity (*n* = 2), DCM patients with KSHV DNA negativity (*n* = 11), and DCM patients with KSHV DNA positivity (*n* = 14). **e** The copies of kshv-miR-K12-1-5p in the plasma samples from non-DCM (*n* = 937) and DCM (*n* = 696) patients. **f** The positivity of KSHV DNA and KSHV DNA copies (**g**) in the plasma from non-DCM (*n* = 937) and DCM patients (*n* = 696). **h** The copies of kshv-miR-K12-1-5p in the plasma from non-DCM with KSHV DNA negativity (*n* = 852), non-DCM with KSHV DNA positivity (*n* = 85), DCM patients with KSHV DNA negativity (*n* = 543), and DCM patients with KSHV DNA positivity (*n* = 153). *p < 0.05

In our previous study, we identified three of the upregulated cardiac miRNAs and seven of the upregulated circulating miRNAs were encoded by KSHV, in which only one, kshv-miR-K12-1-5p, was elevated both in the heart and plasma of patients with DCM.^22^ To independently validate our previous miRNA profile results, we enrolled another cohort of frozen left ventricle samples from 14 healthy donors and 25 recipients of heart transplantation with DCM and end-stage HF (Supplementary Table 1). Results showed that the expression of kshv-miR-K12-1-5p and kshv-miR-K12-12-3p were increased, while kshv-miR-K12-6-3p showed no significant difference, in the heart tissues from patients with DCM compared to that in the control subjects (Fig. 1a and Supplementary Fig. 1a, b). Then we speculated the discrepancy of KSHV load in patients with DCM compared with the control subjects. Using quantitative PCR assays, the KSHV DNA was detectable in 14 of 25 heart tissues from patients with DCM but only 2 of 14 heart tissues from healthy donors (56.0% versus 14.3%, *p* < 0.05, Fig. 1b). The KSHV DNA load was also increased in the heart from patients with DCM in comparison with healthy donors (1016 versus 29 copies/10^5^ cells, *p* < 0.05, Fig. 1c). Especially, kshv-miR-K12-1-5p level was even higher in KSHV DNA positivity DCM patients (Fig. 1d). Using whole-exome sequencing, we identified 10 of these 25 DCM patients carrying the pathogenic variants on the known causal genes for DCM, which were noted as genetic DCM (Supplementary Table 2). We found there was no significant difference in kshv-miR-K12-1-5p levels or KSHV DNA copies between the heart samples from non-genetic and genetic DCM (Supplementary Fig. 1c, d).

The miRNA profile also showed that the cardiac levels of kshv-miR-K12-1-5p and kshv-miR-K12-6-3p were positively correlated with its level in plasma samples, while there was no correlation between the cardiac and plasma levels of kshv-miR-K12-12-3p (Supplementary Fig. 1e). Considering the cardiac level and the correlation between the cardiac and plasma levels of KSHV-encoded miRNAs, circulating kshv-miR-K12-1-5p was chosen to be a reliable surrogate marker for cardiac status. The levels of kshv-miR-K12-1-5p were then measured in a second set of plasma samples from 696 patients with DCM and 937 patients without DCM (non-DCM) (Table 1). Our results showed a 2.36-fold increase in kshv-miR-K12-1-5p level in patients with DCM compared to non-DCM subjects (Fig. 1e). Meanwhile, in plasma samples cohort, a KSHV infection rate of 22.0% (153 of 696) in DCM patients was significantly higher than in non-DCM people, 9.1% (85 of 937) (Fig. 1f). Most of the patients came from Hubei province and there was no significant difference in the regional distribution among the total, DCM, and non-DCM populations (Supplementary Fig. 2). As well, the KSHV virus titers were significantly higher in DCM patients than in non-DCM people (168 versus 14 copies/mL plasma, *p* < 0.05, Fig. 1g). There was a 15.06-fold increase in kshv-miR-K12-1-5p level in DCM patients with KSHV DNA seropositivity compared to non-DCM subjects with seronegativity, as well as a 2.15-fold increase compared to non-DCM subjects with seropositivity (Fig. 1h). The clinical characteristics were shown in Supplementary Table 3. To address the specificity of kshv-miR-K12-1-5p, we validated the real-time PCR products of kshv-miR-K12-1-5p in plasma detection using Sanger sequencing. As shown in Supplementary Fig. 3a, the sequence of real-time PCR products was exactly the same as kshv-miR-K12-1-5p (MIMAT0002182) in the miRBase database.^28^ And kshv-miR-K12-1-5p was uniquely expressed in KSHV-infected BCBL-1 cell, but not in Molt-4 (neither KSHV nor EBV-infected) or Raji (EBV-infected) cells (Supplementary Fig. 3b). In addition, we collected the corresponding heart, peripheral blood mononuclear cell and plasma samples from 5 patients with DCM, and detected the KSHV DNA using PCR, respectively. The results of KSHV DNA detection in the heart samples were not always consistent with that in peripheral blood mononuclear cell and plasma samples from the same patient (Supplementary Fig. 3c). Although most patients showed the same pattern of KSHV DNA detection in the heart and plasma samples, one patient (patient 3) with cardiac KSHV DNA positivity was seronegativity in the plasma (Supplementary Fig. 3c). In addition, the plasma levels of anti-KSHV IgG were measured in 90 randomly chosen participants from the plasma cohort and showed 92.2% concordance with the KSHV DNA detection (Supplementary Table 4). As the clinical variables differed significantly between DCM and non-DCM patients, we performed propensity score-matched analysis to minimize the baseline differences: 502 DCM cases were matched at a 1:1 ratio with 502 non-DCM subjects. After this refinement, baseline characteristics were comparable between the two groups (Supplementary Table 5). Consistently, a significant increase, in the copies of kshv-miR-K12-1-5p, the positivity of KSHV DNA and KSHV DNA copies, respectively, was still found in the plasma samples from DCM patients compared to non-DCM subjects (Supplementary Fig. 4).

**Table 1:** Clinical characteristics of patients included in the plasma KSHV DNA and miRNA detection

Variables	Non-DCM (*n* = 937)	DCM (*n* = 696)	*p* value
Age, years	59 ± 12	56 ± 14	0.010^a^
Female, *n* (%)	533 (56.9%)	247 (35.3%)	<0.001^b^
Smoking, *n* (%)	252 (26.9%)	243 (35.1%)	0.001^b^
Heart rate, beats/min	78 ± 14	85 ± 18	<0.001^a^
SBP, mmHg	133 ± 22	125 ± 22	<0.001^a^
DBP, mmHg	80 ± 14	80 ± 15	0.108^a^
LVEF, %	60.93 ± 8.99	34.06 ± 11.89	<0.001^a^
LVEDD, mm	47.02 ± 4.78	65.06 ± 8.33	<0.001^a^
NYHA class II, III, or IV	260 (27.7%)	652 (93.7%)	<0.001^b^
Hypertension	450 (48.0%)	260 (37.4%)	<0.001^b^
Type 2 diabetes	151 (16.1%)	104 (14.9%)	0.519^b^
Hyperlipidemia	110 (11.7%)	61 (8.8%)	0.052^b^

Age, heart rate, SBP, DBP, LVEF, and LVEDD are given as mean ± SD, and other values as the number of individuals (*n*) with percentage (*n*/*N*) in parentheses

*DCM* dilated cardiomyopathy, *HF* heart failure, *SBP* systolic blood pressure, *DBP* diastolic blood pressure, *LVEF* left ventricular ejection fraction, *LVEDD* left ventricular end diastolic diameter

^a^By the Mann–Whitney *U*-test

^b^By the *χ*^2^ test or Fisher’s exact test

These data supported that KSHV prevalence and its miRNA, kshv-miR-K12-1-5p, were both increased in heart and plasma samples from DCM patients.

### Increased kshv-miR-K12-1-5p level and KSHV prevalence were associated with poor prognosis of DCM

**Fig. 2:**
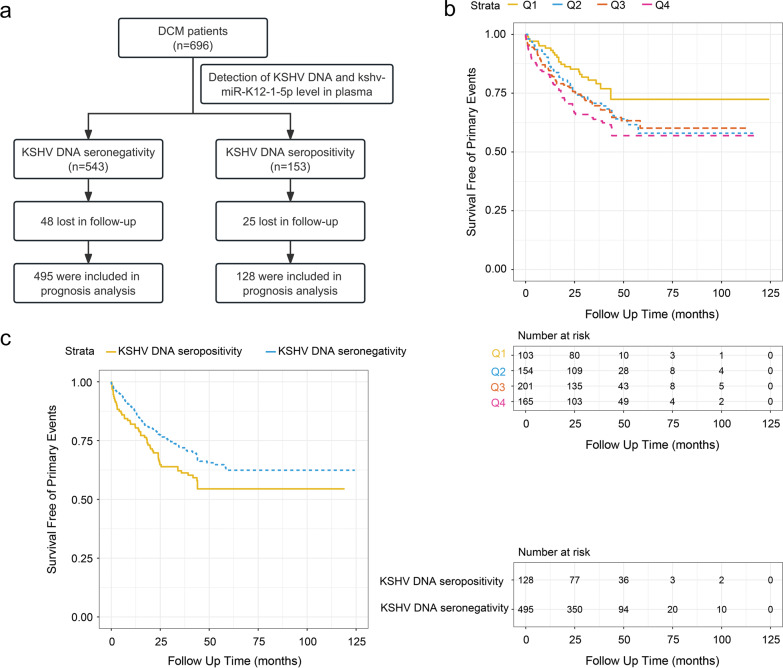
Increased kshv-miR-K12-1-5p level and KSHV prevalence were associated with poor prognosis of DCM. **a** A flowchart of the plasma cohort study. **b** Survival curves for the primary endpoint across kshv-miR-K12-1-5p quartiles. Q1, the first (lowest) quartile; Q2, the second quartile; Q3, the third quartile; Q4, the fourth (highest) quartile. **c** Survival curves for the primary endpoint for DCM patients with KSHV DNA seronegativity or seropositivity

Based on their plasma levels of kshv-miR-K12-1-5p, the patients were divided into quartiles (Supplementary Table 6). Using the logistic regression model, we found that higher plasma levels of kshv-miR-K12-1-5p were associated with increased odds of DCM (*p* for trend <0.001, Supplementary Table 7). In addition, these DCM patients were followed up for a median duration of 35 months (interquartile range: 19–47 months), and a total of 202 DCM patients (29.0%) reached the primary end point. Because 48 of seronegativity group and 25 of seropositivity group were lost in follow-up, 495 of DCM patients with KSHV DNA seronegativity and 128 with KSHV DNA seronegativity were included in prognosis analysis (Fig. 2a). Multivariable analyses showed that higher kshv-miR-K12-1-5p levels were strongly associated with increased risks of cardiac death or heart transplantation in DCM patients (Q4 versus Q1, adjusted hazard ratio (HR_adj_) 2.00, 95% confidence interval (CI) 1.24–3.25; *p* = 0.005, Supplementary Table 8). The survival curves comparing the freedom from the primary composite endpoint were shown in Fig. 2b. In addition, we analyzed the prognosis of DCM patients with KSHV DNA seropositivity or seronegativity. As shown in Fig. 2c, the risk of the individual end point of death from cardiovascular causes or heart transplantation was increased among DCM patients with the KSHV DNA seropositivity during follow-up (HR_adj_ 1.38, 95% CI 1.01–1.90; *p* = 0.043).

These results indicated a potential association between increased kshv-miR-K12-1-5p level and KSHV prevalence with poor prognosis of DCM.

### kshv-miR-K12-1-5p might partially originate from KSHV-infected cardiac endothelial cells

**Fig. 3:**
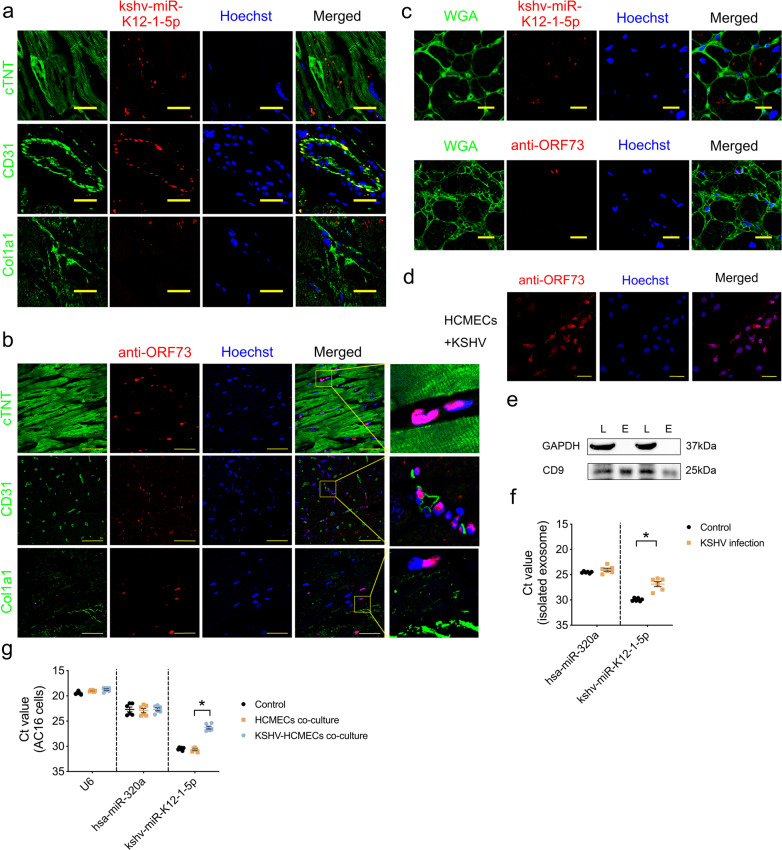
kshv-miR-K12-1-5p might partially originate from KSHV-infected cardiac endothelial cells. **a** Representative images of fluorescence in situ hybridization of kshv-miR-K12-1-5p (red) as well as markers of cardiomyocytes (cTNT), endothelial cells (CD31) or fibroblasts (Col1a1). Scale bar = 25 µm. **b** Representative images of immunofluorescence staining of KSHV ORF73 (red). Scale bar = 50 µm. **c** Representative images of fluorescence in situ hybridization of kshv-miR-K12-1-5p (red, up) or immunofluorescence staining of KSHV ORF73 (red, down) as well as WGA membrane staining (green) in KSHV positive DCM heart. Scale bar = 20 µm. **d** Representative images of immunofluorescence staining of KSHV ORF73 (red) in KSHV-infected HCMECs. Scale bar = 50 µm. **e** The expression of GAPDH and exosome marker CD9 detected by Western blot. L cell lysate, E exosome. **f** Ct value of kshv-miR-K12-1-5p and miR-320a in isolated exosome extracted from culture media of HCMECs with or without KSHV infection detected by RT-PCR (*n* = 6 each). Lower Ct value indicates a higher initial nucleic acid concentration. **g** Ct value of U6, miR-320a, and kshv-miR-K12-1-5p in RNA extracted from AC16 cells (control), AC16 cells co-cultured with HCMECs, and AC16 cells co-cultured with KSHV-infected HCMECs (KSHV-HCMECs) (*n* = 6 each). *p < 0.05

To investigate the possible cellular origin of the cardiac kshv-miR-K12-1-5p, we performed fluorescence in situ hybridization (FISH) of kshv-miR-K12-1-5p in KSHV positive DCM samples, and found that kshv-miR-K12-1-5p was mainly expressed in endothelial cells, while it could be also detected in cardiomyocytes (Fig. 3a). Furthermore, we noticed that KSHV itself was exclusively detectable in CD31-positive endothelial cells but not in cardiomyocytes (Fig. 3b and Supplementary Fig. 5), thus the cardiomyocyte-localized kshv-miR-K12-1-5p might originate from endothelial cells infected by KSHV. Consistently, the kshv-miR-K12-1-5p FISH or KSHV immunofluorescence staining combined with wheat germ agglutinin (WGA) membrane staining showed that the kshv-miR-K12-1-5p could be detected inside of cardiomyocytes as well as non-myocytes, while the KSHV was detectable outside of cardiomyocytes (Fig. 3c). We also performed the kshv-miR-K12-1-5p FISH and KSHV immunofluorescence staining in KSHV negative DCM heart, normal heart with KSHV positivity and normal heart with KSHV negativity. The results showed a small amount of fluorescence signal could be detected in normal heart with KSHV positivity, while neither the signal of kshv-miR-K12-1-5p nor KSHV could be detected in KSHV-negative DCM and normal heart (Supplementary Fig. 6).

It has been reported previously that KSHV-infected cells could release extracellular vesicles containing viral miRNAs with high concentrations in culture media and in patients.^29,30^ We infected human cardiac microvascular endothelial cells (HCMECs) with KSHV in vitro, evidenced by anti-KSHV immunofluorescence staining (Fig. 3d). Besides, KSHV could infect HUVEC (human endothelial cell line), but not human induced pluripotent stem cell-derived cardiomyocyte (hiPSC-derived CM), AC16 (human cardiomyocyte cell line) or HL-1 (mouse cardiomyocyte cell line) (Supplementary Fig. 7). The exosome, characterized by inclusion of CD9 and exclusion of GAPDH, was isolated from culture media of HCMECs with or without KSHV infection (Fig. 3e). Consistently, the level of KSHV-encoded kshv-miR-K12-1-5p was higher in exosome fraction from culture media of KSHV-infected HCMECs (KSHV-HCMECs), evidenced by lower Ct value of kshv-miR-K12-1-5p, while the release of host-encoded miRNA such as miR-320a was unaffected by KSHV infection (Fig. 3f). Moreover, after co-cultured with KSHV-HCMECs, the kshv-miR-K12-1-5p was detectable in AC16 cells (Fig. 3g), indicating the transfer of kshv-miR-K12-1-5p from infected-endothelial cells to cardiomyocytes.

These data suggested that the cardiomyocyte-localized kshv-miR-K12-1-5p might partially originate from KSHV-infected cardiac endothelial cells.

### kshv-miR-K12-1-5p targeted type I IFN signaling pathway

**Fig. 4:**
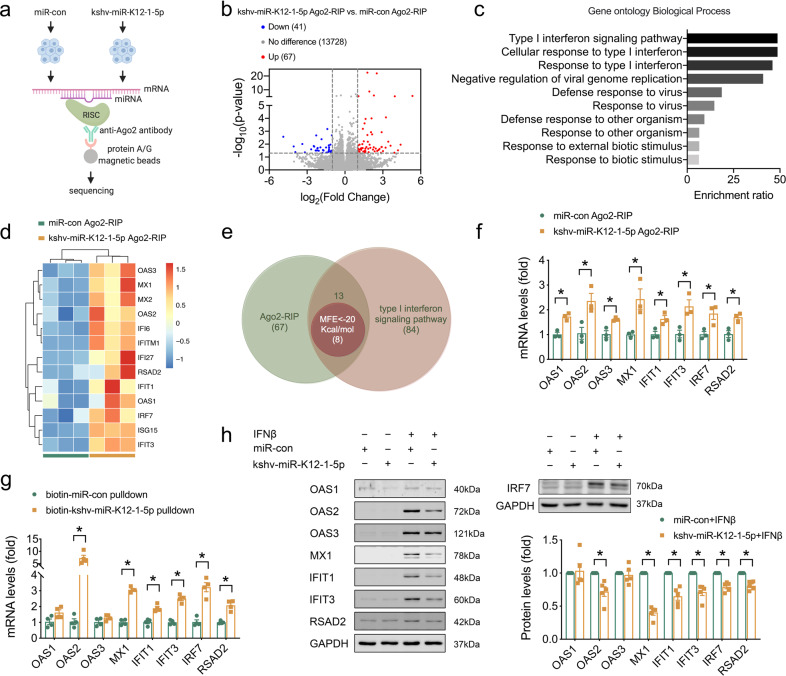
kshv-miR-K12-1-5p targeted IFN signaling pathway. **a** Schematic diagram of Ago2 RNA immunoprecipitation and sequencing (Ago2- RIP seq). **b** Volcano plot of Ago2-RIP seq profile. The upregulated and downregulated genes were indicated by red and blue color codes, respectively, following a filtering criterion as fold change>2 and *p* < 0.05. **c** Gene ontology analyses of the upregulated 67 genes. **d** Heat map of the upregulated genes in type I IFN signaling pathway. **e** Venn diagram showing the overlap number of candidate target genes of kshv-miR-K12-1-5p identified by Ago2-RIP seq, type I IFN signaling pathway and RNAhybrid. **f** The RNA levels pulled down by anti-Ago2 antibody in hiPSC-derived CM (*n* = 3 each). **g** The RNA levels pulled down by biotin-labeled kshv-miR-K12-1-5p in AC16 cells (*n* = 4 each). **h** The expression of candidate target genes detected by Western blot in AC16 cells with kshv-miR-K12-1-5p transfection followed by IFNβ treatment (*n* = 5 each). *p < 0.05

Next, we explored the potential effect of kshv-miR-K12-1-5p on DCM. To understand the underlying molecular mechanisms of kshv-miR-K12-1-5p, we performed RNA immunoprecipitation sequencing (RIP-seq) using anti-Argonaute2 (Ago2) antibody to identify the potential targets of kshv-miR-K12-1-5p in AC16 cells (Fig. 4a). Using a cutoff of fold change>2 and *p* < 0.05, we identified 67 mRNAs that showed increased association with the Ago2 protein after kshv-miR-K12-1-5p transfection (Fig. 4b), suggesting that kshv-miR-K12-1-5p might enhance these mRNAs packaged into RNA-induced silencing complex. These 67 genes were selected as a gene set for the gene ontology analyses, of which the type I IFN signaling pathway was the most enriched pathway, including 13 genes [OAS1, OAS2, OAS3, MX1, MX2, interferon alpha inducible protein (IFI) 6, IFI27, interferon induced protein with tetratricopeptide repeats (IFIT) 1, IFIT3, interferon induced transmembrane protein 1 (IFITM1), IRF7, ISG15, and radical S-adenosyl methionine domain containing 2 (RSAD2)] (Fig. 4c, d). Subsequently, the online computational tool, RNAhybrid, was used to screen the potential binding sites of kshv-miR-K12-1-5p on these 13 mRNA sequences, among which 8 candidate genes-OAS1, OAS2, OAS3, MX1, IFIT1, IFIT3, IRF7, and RSAD2 were identified as the potential targets of kshv-miR-K12-1-5p, following a filtering criterion as minimum free energy (MFE) lower than −20 Kcal/mol (Fig. 4e). These candidate genes were substantiated by Ago2 RIP in hiPSC-derived CM. Consistent with the data obtained in AC16 cells, the identified type I IFN genes showed increased association with the Ago2 protein after kshv-miR-K12-1-5p transfection in hiPSC-derived CM (Fig. 4f). Using biotin-labeled miRNA pulldown assay, 6 of 8 type I IFN genes we identified were pulled down by biotin-labeled kshv-miR-K12-1-5p (Fig. 4g), supporting these genes as the targets of kshv-miR-K12-1-5p. Meanwhile, transfection of kshv-miR-K12-1-5p with or without biotin labeling did not affect the IFNβ1 expression in AC16 cells (Supplementary Fig. 8a, b).

Western blots were performed to validate the effects of kshv-miR-K12-1-5p on these genes. As shown in Fig. 4h and Supplementary Fig. 8c, IFNβ significantly activated the type I IFN signaling pathway in AC16 cells, while kshv-miR-K12-1-5p reduced the expression of most of IFNβ1 downstream genes without influencing itself level. Furthermore, other viral miRNA (ebv-miR-BHRF1-2-5p or hcmv-miR-UL112-3p, respectively) had no effect on these type I IFN genes but increased the level of their previously reported target (IL1R1^31^ or TLR2^32^, respectively) pulled down by anti-Ago2 antibody (Supplementary Fig. 9a), suggesting an unique association between these type I IFN genes and kshv-miR-K12-1-5p. Next, the luciferase reporter containing the potential binding sites in 3′-UTR of OAS2, IFIT1, IFIT3 or RSAD2, as well as in 5′-UTR of MX1 or IRF7, was co-transfected with kshv-miR-K12-1-5p mimics into HEK293 cells and all these luciferase activities were inhibited by kshv-miR-K12-1-5p (Supplementary Fig. 9b, c).

These results supported that kshv-miR-K12-1-5p disrupted the type I IFN signaling pathway via directly targeting its downstream genes.

### kshv-miR-K12-1-5p weakened the anti-viral effects of IFNβ

We determined the plasma level of IFNβ, one of the most studied type I IFNs, in 122 non-DCM patients (of 937) and 123 DCM patients (of 696) using ELISA measurement. The plasma IFNβ level in DCM patients was much higher than non-DCM subjects (Supplementary Fig. 10a). Moreover, the IFNβ level was positively correlated with the levels of circulating kshv-miR-K12-1-5p (Supplementary Fig. 10b). Consistently, the level of IFNβ in cellular supernatant was increased after CVB3 infection, while kshv-miR-K12-1-5p further increased IFNβ level (Supplementary Fig. 10c). However, the levels of IFNβ1 downstream genes (IFIT1, IFIT3, RSAD2 and IRF7) were negatively correlated with kshv-miR-K12-1-5p expression in DCM hearts (Supplementary Fig. 11), suggesting that type I IFN signaling pathway was disrupted not only in local hearts, but also systemically, in DCM patients with higher kshv-miR-K12-1-5p levels.

**Fig. 5:**
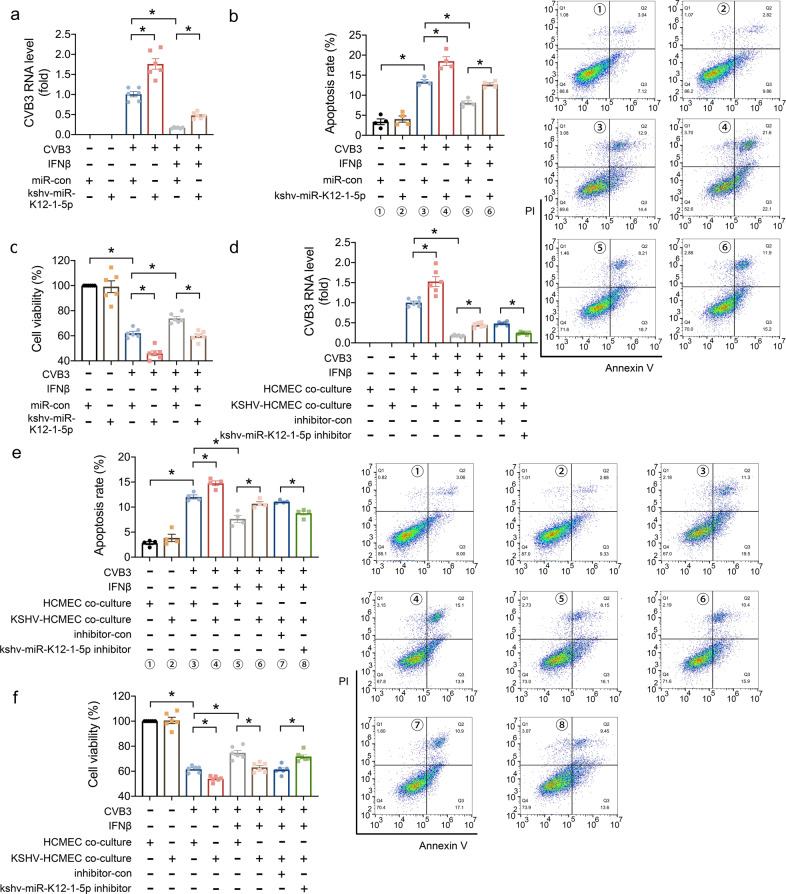
kshv-miR-K12-1-5p weakened the anti-viral effects of IFNβ. AC16 cells were transfected with kshv-miR-K12-1-5p mimics or miR-con, followed by CVB3 infection and IFNβ treatment. Twenty-four hours later, the CVB3 RNA level in cells was detected by RT-PCR (**a**), the apoptotic cells were detected by flow cytometry (**b**), and the cell viability were detected by CCK8 (**c**). Twenty-four hours after co-cultured with KSHV infected-HCMECs (KSHV-HCMECs), the AC16 cells were transfected with kshv-miR-K12-1-5p inhibitor followed by CVB3 and IFNβ treatment. Another 24 h later, the CVB3 RNA level in cells was detected by RT-PCR (**d**), the apoptotic cells were detected by flow cytometry (**e**), and the cell viability were detected by CCK8 (**f**). n = 6 for **a**, **c**, **d**, **f**; *n* = 4 for **b**, **e**. *p < 0.05

Furthermore, we tested the effects of kshv-miR-K12-1-5p on the anti-viral function of type I IFN signaling pathway in vitro. The kshv-miR-K12-1-5p mimics or negative control (miR-con) were transfected to AC16 cells, following a known cardiotropic virus (CVB3) and IFNβ treatment. The results showed that kshv-miR-K12-1-5p had no effect on apoptosis and cell viability of AC16 cells without CVB3 infection, while it increased the CVB3 levels, as well as aggravated the apoptosis and viability loss of AC16 cells induced by CVB3 (Fig. 5a–c). IFNβ treatment reduced the CVB3 RNA level and cell apoptosis induced by CVB3, while kshv-miR-K12-1-5p transfection weakened the anti-viral effects of IFNβ (Fig. 5a–c). Some downstream genes of IFNβ were increased in AC16 cells after CVB3 infection and IFNβ treatment, while kshv-miR-K12-1-5p transfection inhibited most of these genes’ expression (Supplementary Fig. 12). We also investigated the effects of kshv-miR-K12-1-5p on virus replication and cell apoptosis in hiPSC-derived CM. Consistently, kshv-miR-K12-1-5p transfection had no influence on apoptosis and myocytes contractility under normal condition, while it reduced the anti-viral effects of IFNβ in hiPSC-derived CM (Supplementary Fig. 13).

In addition, 24 h after co-cultured with KSHV infected-HCMECs (KSHV-HCMECs), the AC16 cells were transfected with kshv-miR-K12-1-5p inhibitor followed by CVB3 and IFNβ treatment. The results showed that KSHV-HCMECs co-culture increased the CVB3 replication, as well as the apoptosis and viability loss induced by CVB3 in AC16 cells (Fig. 5d–f). And KSHV-HCMECs co-culture reduced the anti-viral effects of IFNβ reflected by CVB3 RNA level, while kshv-miR-K12-1-5p inhibitor blocked the influence of co-culture (Fig. 5d–f).

These results indicated kshv-miR-K12-1-5p weakened the anti-viral effects of IFNβ in vitro.

### kshv-miR-K12-1-5p aggravated cardiotropic viruses-induced cardiac inflammatory infiltration and dysfunction

**Fig. 6:**
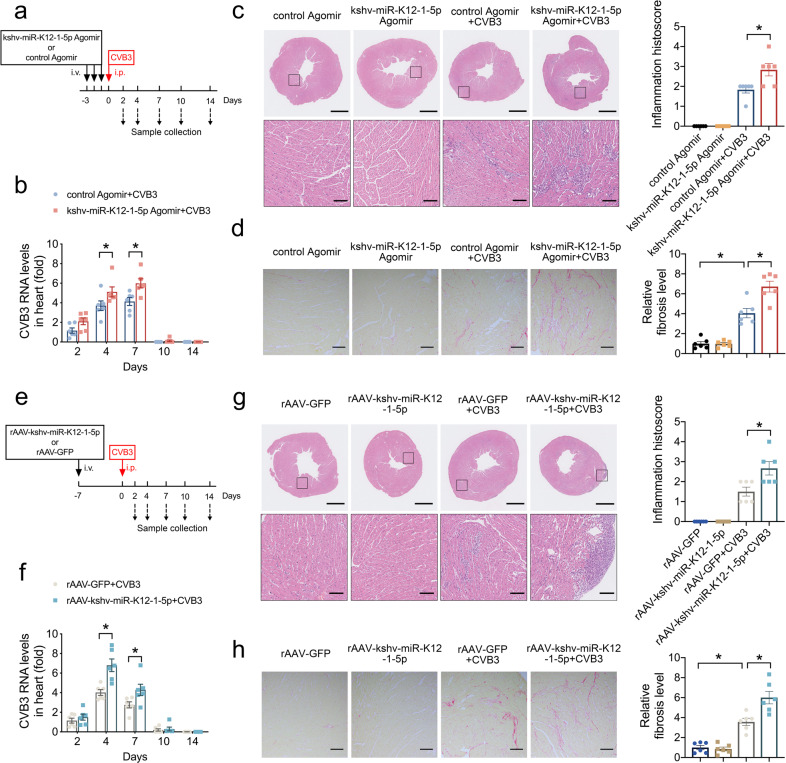
kshv-miR-K12-1-5p aggravated cardiotropic viruses-induced cardiac inflammatory infiltration and dysfunction. **a** Schema of the experimental setup for overexpressing kshv-miR-K12-1-5p via agomiR. i.v. tail vein injection, i.p. intraperitoneal injection. **b** The CVB3 RNA levels detected by RT-PCR in the heart tissues at different time points. **c** HE staining of representative heart tissue sections (left) and the inflammation histoscore calculated based on HE staining (right) on day 7 post-infection. Scale bar (up) = 1 mm. Scale bar (down) = 100 µm. **d** Sirius red staining of representative heart tissue sections (left) and statistical graph (right) on day 14 post-infection. Scale bar = 100 µm. **e** Schema of the experimental setup for overexpressing kshv-miR-K12-1-5p via rAAV. i.v. tail vein injection, i.p. intraperitoneal injection. **f** The CVB3 RNA levels detected by RT-PCR in the heart tissues at different time points. **g** HE staining of representative heart tissue sections (left) and the inflammation histoscore calculated based on HE staining (right) on day 7 post-injection. Scale bar (up) = 1 mm. Scale bar (down) = 100 µm. **h** Sirius red staining of representative heart tissue sections (left) and statistical graph (right) on day 14 post-injection. Scale bar = 100 µm. *n* = 6 each. *p < 0.05

We detected the cardiotropic viruses reported previously in DCM hearts,^18,33^ finding that the genomes of other viruses, such as CVB3, PB19 and HHV6, were detected in 8 of 14 DCM hearts with KSHV DNA positivity and 3 of 11 DCM hearts with KSHV DNA negativity (Supplementary Table 9). The data suggested that the DCM hearts with KSHV DNA positivity seemed to be more susceptible to other cardiotropic viruses. Therefore, we used two models of kshv-miR-K12-1-5p overexpression followed by known infection of cardiotropic viruses in mice (Fig. 6 and Supplementary Figs. 14 and 15). As a method to express kshv-miR-K12-1-5p in the mouse heart, we injected kshv-miR-K12-1-5p agomiR or a control agomiR for 3 consecutive days prior to CVB3 infection, which was performed one day after the last agomiR injection (Fig. 6a). The mice were killed to detected CVB3 RNA levels in the heart tissues at different time point. The kshv-miR-K12-1-5p agomiR increased the CVB3 replication in the heart at days 4 and 7 post-infection (Fig. 6b). In addition, kshv-miR-K12-1-5p agomiR increased the infiltration by inflammatory cells in the heart tissues and serum levels of inflammatory cytokines at day 7 (Fig. 6c and Supplementary Fig. 14a). At day 14, we also observed that kshv-miR-K12-1-5p agomiR aggravated the fibrosis level in the heart tissues, as well as cardiac output decrease and diastolic dysfunction (Fig. 6d, Supplementary Fig. 15a–c, and Supplementary Table 10).

A rAAV-mediated cardiac enriched kshv-miR-K12-1-5p overexpression approach was next assessed (Fig. 6e). Similar to our findings with agomiR, rAAV-kshv-miR-K12-1-5p increased the CVB3 RNA levels in the heart at days 4 and 7, the inflammatory infiltration in the heart and cytokines in plasma at day 7, as well as the fibrosis area in the heart tissues at day 14 (Fig. 6f–h and Supplementary Fig. 14b). Correspondingly, rAAV-kshv-miR-K12-1-5p exacerbated the cardiac output decrease and diastolic dysfunction induced by CVB3 infection at day 14 (Supplementary Fig. 15d–f and Supplementary Table 11). Consistently, both approaches mediated kshv-miR-K12-1-5p overexpression increased the encephalomyocarditis virus (EMCV) replication in the heart and serum levels of inflammatory cytokines (Supplementary Fig. 14c–f). Furthermore, the protein level of IFNβ1 downstream genes was measured in two mice models of kshv-miR-K12-1-5p overexpression followed by 7-day CVB3 infection. Consistent results showed that the expression of IFIT1, RSAD2 and IRF7 were induced in CVB3-infected mouse hearts, while overexpression of kshv-miR-K12-1-5p inhibited these responses (Supplementary Fig. 16a, b).

To investigate the role of type I IFN signaling pathway in kshv-miR-K12-1-5p effects, the anti-IFNAR neutralizing monoclonal antibody was used before CVB3 infection (Supplementary Fig. 17a). The mice were killed on day 5 after CVB3 infection, because 50% of mice with anti-IFNAR and CVB3 treatments suffered death at this time (Supplementary Fig. 17b). As shown in Supplementary Fig. 17c, anti-IFNAR treatment significantly increased the CVB3 replication in the heart, while there was no difference in CVB3 RNA levels between rAAV-kshv-miR-K12-1-5p+anti-IFNAR and rAAV-GFP + anti-IFNAR group. In addition, anti-IFNAR treatment increased the infiltration by inflammatory cells in the heart tissues and serum level of inflammatory cytokines, while rAAV-kshv-miR-K12-1-5p could not aggravate CVB3 induced inflammatory infiltration after type I IFN signaling was blocked (Supplementary Fig. 17d, e). These data suggested that kshv-miR-K12-1-5p acted on IFN signaling pathway to enhance the CVB3 induced cardiac inflammatory response.

To observe the phenotype of kshv-miR-K12-1-5p alone in a much longer term, mice were injected with rAAV-GFP or rAAV-kshv-miR-K12-1-5p, respectively, for 6 weeks. There was no significant difference in cardiac structure and function measured by cardiac echocardiography and hemodynamic analysis (Supplementary Fig. 18a–c and Supplementary Table 12). Without additional infection, the rAAV-kshv-miR-K12-1-5p treatment could not affect the inflammation or fibrosis in the heart (Supplementary Fig. 18d). Using transmission electron microscope and atomic force microscopy, we did not observe significant structural disorganization of sarcomeres in mouse heart with kshv-miR-K12-1-5p overexpression (Supplementary Fig. 18e, f). Consistently, sarcomere shortening detection showed that rAAV-kshv-miR-K12-1-5p under normal conditions did not alter cardiomyocyte function (Supplementary Fig. 18g). In addition, we overexpressed kshv-miR-K12-1-5p using rAAV in transverse aortic constriction (TAC)-induced heart failure model and found that kshv-miR-K12-1-5p showed no effect on cardiac function in TAC-induced heart failure mice on the condition that IFNβ level was unchanged (Supplementary Fig. 19 and Supplementary Table 13). These data suggested the effects of kshv-miR-K12-1-5p on cardiac function rely on the type I IFN signaling pathway.

Our study indicated that kshv-miR-K12-1-5p aggravated cardiotropic viruses-induced cardiac inflammatory infiltration and dysfunction via inhibiting IFN signaling pathway.

## Discussion

In the present study, we found that KSHV prevalence and its miRNA, kshv-miR-K12-1-5p, were both increased and associated with poor prognosis of DCM. Human cardiac endothelial cells infected by KSHV could release kshv-miR-K12-1-5p, which would disrupt type I IFN signaling pathway in cardiomyocytes. Our results suggested that KSHV infection was a risk factor for DCM via secreting a microRNA that suppresses type I IFN signaling and might increase subsequent infection with known cardiotropic viruses (Supplementary Fig. 20).

Viral infection is the most common cause of myocarditis, which might lead to the development of DCM and HF, sometimes years later.^7^ In addition, viral persistence in the heart was reported to be associated with progressive cardiac dysfunction.^6^ To date, many viruses have been found in the heart of DCM patients, such as adenovirus, enterovirus, PB19, and HHV6.^9^ In the present study, we detected KSHV DNA in the heart tissues and its prevalence was significantly increased in DCM patients. In addition, we detected the cardiotropic viruses reported previously in DCM hearts and found that the DCM hearts with KSHV DNA positivity seemed to be more susceptible to other cardiotropic viruses, supporting our conclusion that KSHV infection was a risk factor for DCM via increasing subsequent infection with known cardiotropic viruses.

Although KSHV itself was exclusively detectable in endothelial cells in DCM heart samples, kshv-miR-K12-1-5p could be detected in both endothelial cells and cardiomyocytes. Consistently, it has been reported that high concentrations of viral miRNAs could be released by KSHV-infected cells into culture media via extracellular vesicles.^30^ KSHV miRNAs could also be detectable in exosome fraction isolated from plasma of patients with KS.^29^ In vitro, we observed KSHV-infected endothelial cells released kshv-miR-K12-1-5p into culture media via exosome, which was taken in co-cultured cardiomyocytes, suggesting that kshv-miR-K12-1-5p may partially originate from cardiac endothelial cells.

Viruses utilize functional RNA interference by expressing their own miRNAs. In addition to cellular miRNAs, various viral miRNAs with aberrant expression have been identified in patients and were found to contribute to the progression of diseases.^21,27^ In our study, we found obviously increased levels of kshv-miR-K12-1-5p in the heart and plasma samples of DCM patients. Using Ago2 RIP-seq, we found that kshv-miR-K12-1-5p inhibited the expression of a number of antiviral proteins such as MX1, OAS, and IRFs, which are the downstream of type I IFN. As a crucial antiviral member, type I IFN signaling pathway exerts various effects including innate immune antiviral action and modulation of cytokine production.^14,34^ The inhibiting of type I IFN signaling pathway by kshv-miR-K12-1-5p emphasized its effects on resisting viral clearance.

In addition, kshv-miR-K12-1-5p had no influence on IFNβ level without CVB3. However, the level of IFNβ in cellular supernatant was increased after CVB3 infection, while kshv-miR-K12-1-5p further increased IFNβ level which was consistent to the circulating level of IFNβ in patients with DCM. We speculated that kshv-miR-K12-1-5p could not influence the expression of IFNβ directly. Upon CVB3 infection, IFNβ level was increased and its downstream signals were activated to clear CVB3. However, kshv-miR-K12-1-5p inhibited the downstream signals and impaired the clearance of CVB3, then the accumulated CVB3 in turn induced more IFNβ. Almost all cells in the body can produce IFNβ1, and this usually occurs in response to the stimulation of receptors known as pattern recognition receptors (PRRs) by microbial products.^35,36^ Immune cells, such as macrophage and plasmacytoid dendritic cells, could also contribute to the increased circulating IFNβ1 level in response to viruses.^35,37^ The increase in circulating IFNβ1 might be triggered by the systemic microenvironment feedback mechanisms. Interestingly, we found that some non-DCM patients had high levels of kshv-miR-K12-1-5p in their hearts, which might require extended follow-up period to observe whether they would develop DCM.

There are some limitations in this study. Considering potential virus clearance and infection, it would be helpful to test the KSHV positivity status in follow up studies. Besides, we failed to investigate the function of KSHV in vivo. Our findings revealed a novel virus that might be associated with DCM progression in the heart. However, because the natural infection by KSHV is limited to human, most experiments transmitting this virus into other animal species such as mice have failed.^38^ So, we used two models of kshv-miR-K12-1-5p overexpression followed by known cardiotropic viruses infection in mice to explore the roles of kshv-miR-K12-1-5p in vivo. Our study indicated that kshv-miR-K12-1-5p aggravated known cardiotropic viruses-induced cardiac dysfunction and inflammatory infiltration through inhibiting IFNβ signaling pathway together with previous results. Thus, KSHV infection is a risk factor for DCM via secreting a microRNA that suppresses type I IFN signaling and might increases subsequent infection with known cardiotropic viruses.

Most HIV-negative and KSHV-infected individuals never develop KS.^23^ However, KSHV, like the majority of herpesviruses, is involved in a lifelong and persistent infection in immunocompetent hosts, and is associated with an array of derived miRNAs.^39^ It has been reported the KSHV-encoded miRNAs are involved in sepsis.^27^ Here we found that KSHV-encoded miRNA was associated with the progression of DCM. Thus, KSHV infection status, especially in individuals with underlying cardiovascular diseases, might require more attention.

## Materials and methods

More detailed procedures are provided in the Supplementary Methods.

### Study population

The human heart and plasma samples were collected at Tongji Hospital (Wuhan, China). The study protocol was approved by the Ethics Review Board of Tongji Hospital and Tongji Medical College. The subjects recruited in the study, or immediate family members in the case of incapacity, provided signed, informed consent. The cohort of heart samples comprised 14 healthy donors and 25 receipts of heart transplantation with DCM and end-stage HF, which were absent from significant primary valvular disease, coronary artery disease or myocarditis (Supplementary Table 1). All samples from these 25 patients with DCM were obtained during transplantation before they received the normal hearts and immunosuppressive therapy. The plasma sample cohort comprised 696 patients with DCM and 937 patients without DCM according to the ACC/AHA guidelines (Table 1). Criteria for the diagnosis of DCM were ejection fraction>2 SD below and LV end-diastolic diameter>2 SD above the mean corrected for age and sex.^40^ The exclusion criteria were: severe primary valvular heart disease, congenital heart disease, acute myocardial infarction, or unstable angina within 1 month before admission, dilated phase of hypertrophic cardiomyopathy or refusal to participate in the follow-up. From April 2008 to August 2018, 696 patients with DCM and 937 patients without DCM were recruited from Cardiology Division of Tongji Hospital. These 696 patients with DCM (derived from the clinical trial NCT03461107, https://clinicaltrials.gov.) were followed up periodically through telephone interviews or visits. The primary end point was a composite of death from cardiovascular causes or heart transplantation.

### Detection of KSHV DNA and other viral genomes

DNA was extracted from the heart or 250 µL of separated plasma samples using the E.Z.N.A Viral DNA Kit (Cat#D3892-02, Omega Bio-tek, Norcross, GA) according to the manufacturer’s protocol. KSHV DNA was amplified by TaqMan real-time PCR with KSHV-specific primers as reported previously.^41^ Primers used are listed in Supplementary Table 14. The standard curve of the Ct values obtained from serial dilutions (10 to 10^7^ copies) of the plasmid containing the target sequence was constructed for both KSHV and the human GAPDH gene. The Ct values from the heart or plasma samples were plotted on the standard curves, and the ratio of the number of KSHV genomes per cell or KSHV DNA copies/mL plasma was calculated. A negative result indicated no DNA detection.

Total RNA isolated from the DCM heart tissues was reversely transcribed into cDNA, which served as the template to detect the genomes of CVB3 and H1N1 by real-time PCR. The genomes of other viruses were measured using the DNA isolated from the DCM heart tissues by real-time PCR. Primers used are listed in Supplementary Table 14.

### Argonaute2 RNA immunoprecipitation and sequencing (Ago2-RIP seq)

Twenty-four hours after transfection with kshv-miR-K12-1-5p mimics, ebv-miR-BHRF1-2-5p mimics, hcmv-miR-UL112-3p mimics or miR-con (RiboBio, Guangzhou, China), AC16 cells or hiPSC-derived CM were lysed and immunoprecipitation was performed using an anti-Ago2 antibody followed by protein A/G magnetic beads (Cat#88802, Thermo Scientific, Shanghai, China), as described previously.^42^ The following sequencing was conducted and analyzed by Personalbio (Shanghai, China) using *DESeq* model.^43^ The accession number for the RNA-seq data was GSE201041 in GEO.

### AgomiR mediated kshv-miR-K12-1-5p gain of function

Phosphate buffered saline (PBS)-dissolved kshv-miR-K12-1-5p agomiR or control agomiR (RiboBio) were administered through tail vein injection at a 20 nmol/mice dose for 3 consecutive days. The virus infection was performed the day following the last agomiR administration.

### Recombinant adeno-associated virus (rAAV) mediated kshv-miR-K12-1-5p gain of function

The rAAV system (type 9) was a kind gift from Dr. Xiao Xiao (East China University of Science and Technology, Shanghai, China). The oligonucleotide, designed as 5′-AGCTTATTACAGGAAACTGGGTGTAAGCTTCAAGAGAGCTTACACCCAGTTTCCTGTAATCCGC-3′ (sense), was synthesized and cloned into the plasmid. rAAVs were packaged via triple plasmid co-transfection in HEK293 cells and purified as described previously.^44^ Then, 100 µL of the rAAV-GFP or rAAV-kshv-miR-K12-1-5p virus (1 × 10^11^ virion particles) was administered via the tail vein injection. The virus infection was performed one weeks after rAAV injection. For a long-term experiment, mice were injected with rAAV-GFP or rAAV-kshv-miR-K12-1-5p and killed after 6 weeks.

### Anti-IFNAR administration

Six days after rAAV-GFP or rAAV-kshv-miR-K12-1-5p injection, mice were received a onetime administration of anti-mouse IFNAR-1 blocking monoclonal antibody (mAB) (MAR1-5A3, Cat#I-401, Leinco Technologies). Each mouse received 2.5 mg of anti-IFNAR mAB via intraperitoneal injection 24 h prior to CVB3 infection.

### Animal treatment

All animal experiments complied with the Guide for the Care and Use of Laboratory Animals published by the United States National Institutes of Health and ARRIVE guidelines. The study was approved by the Institutional Animal Research Committee of Tongji Medical College. Mice were purchased from GemPharmatech Co., Ltd. (Nanjing, China). CVB3 was obtained from China Center for Type Culture Collection (Wuhan, China). EMCV was a kind gift from Professor Hong-Bing Shu (Wuhan University, Wuhan, China). For CVB3 infection, BALB/c mice were intraperitoneally injected with 0.1 mL PBS containing 10^5^ 50% tissue culture infectious dose (TCID_50_) CVB3 to induce viral myocarditis. Control mice were intraperitoneally injected with the same volume of PBS. We defined the day of CVB3 inoculation as day 0, and mice were killed on day 2, 4, 7, 10 and 14, respectively. For EMCV infection, C57BL/C mice were intraperitoneally injected with 0.1 mL PBS containing 10^4^ TCID_50_ EMCV to induce viral myocarditis. Control mice were intraperitoneally injected with the same volume of PBS. We defined the day of EMCV inoculation as day 0, and mice were killed on day 2, 4, 7 and 14, respectively.

For TAC-induced heart failure model, eight-week male C57BL/C mice were divided into four groups as follows: rAAV-GFP + Sham, rAAV-kshv-miR-K12-1-5p+Sham, rAAV-GFP + TAC, rAAV-kshv-miR-K12-1-5p+TAC. Pressure overload was induced by TAC in mice as described previously.^45^ The Sham or TAC surgery was performed two weeks after rAAV injection, and mice were killed four weeks after surgery.

### Statistical analysis

Data are reported as the mean ± SEM unless specially mentioned. The Shapiro–Wilk test was used to check the assumption of normality and the Brown–Forsythe test was used to check the equality of variance for each measurement data set. Statistical analyses were then performed with the Student’s *t* test (parametric unpaired or paired, equal variances, two group of analysis), Welch’s *t* test (parametric unpaired, unequal variances, two group of analysis), Mann–Whitney *U*-test (non-parametric unpaired, two group of analysis), one-way analysis of variance (ANOVA) combined with Tukey multiple comparisons test (parametric unpaired, equal variances, more than two groups of analysis), Welch’s ANOVA combined with Dunnett’s T3 multiple comparisons test (parametric unpaired, unequal variances, more than two groups of analysis) and Kruskal–Wallis test with Dunn’s multiple comparisons test (non-parametric unpaired, more than two groups of analysis). Two-way ANOVA combined with Sidak’s multiple comparisons test was used in the in vivo studies when multiple time points were involved. Categorical variables were compared by the *χ*^2^ test or Fisher’s exact test. The odds ratios and 95% confidence intervals were calculated to assess the association between the kshv-miR-K12-1-5p levels and the odds of DCM with the logistic regression model, adjusted for age, gender, ejection fraction, smoking, drinking, hypertension, diabetes, and hyperlipidemia. The proportional hazard assumption was checked by log minus log plot (LML plot) and Schoenfeld residual test. The hazard ratios and 95% confidence intervals were estimated using Cox Proportional-Hazards Regression model, adjusted for age, gender, ejection fraction, smoking, drinking, hypertension, diabetes, and hyperlipidemia. The p-value for trend of hazard ratios across quartiles was calculated using Cox Proportional-Hazards Regression model after merging Q2 and Q3 into one group to satisfy the proportional hazard assumption by Schoenfeld residual test. The Spearman rank correlation coefficient was used to calculate the strength and direction of the association between the IFNβ level and kshv-miR-K12-1-5p level in plasma. All statistics were performed using SPSS 23.0 (IBM Software, Chicago, IL), Stata 16.0 (StataCorp LLC, College Station, TX), or Prism 8.0 (GraphPad Software Inc, San Diego, CA), and differences with *p* < 0.05 were considered significant (two-tailed).

## Supplementary information

Supplementary Material

## Data Availability

The datasets generated during and/or analyzed during the current study are available from the corresponding author on reasonable request.
