# Supplementary material for: A Kaposi’s sarcoma-associated herpes virus-encoded microRNA contributes to dilated cardiomyopathy
Source: Signal Transduct Target Ther. 2023 Jun 9;8:226. doi: 10.1038/s41392-023-01434-3 (PMC10250357; doi:10.1038/s41392-023-01434-3)
Supplement: Supplementary file 1 — Supplementary Material [file 41392_2023_1434_MOESM1_ESM.docx]

Supplementary Materials for

A Kaposi’s sarcoma-associated herpes virus-encoded microRNA contributes to dilated cardiomyopathy

Yanru Zhao^1,2#^, M.D., Ph.D.; Huaping Li^1,2#^, M.D., Ph.D.; Hengzhi Du^1,2#^, M.D.; Zhongwei Yin^1,2^, M.D., Ph.D.; Mengying He^3^, M.D., Ph.D.; Jiahui Fan^1,2^, M.D., Ph.D.; Xiang Nie^1,2^, M.D., Ph.D.; Yang Sun^1,2^, M.D., Ph.D.; Huiying Hou^4^, M.D.; Beibei Dai^1,2^, M.D., Ph.D.; Xudong Zhang^1,2^, M.D., Ph.D.; Yuanyuan Cai^1,2^, M.D.; Kunying Jin^1,2^, M.D.; Nan Ding^1,2^, M.D.; Zheng Wen^1,2^, M.D., Ph.D.; Jiang Chang^5^, Ph.D.; Chen Chen^1,2^*, M.D., Ph.D.; Dao Wen Wang^1,2^*, M.D., Ph.D.

Correspondence to:

Chen Chen, M.D., Ph.D. Email: [chenchen@tjh.tjmu.edu.cn](mailto:chenchen@tjh.tjmu.edu.cn)

Dao Wen Wang, M.D., Ph.D. Email: dwwang@tjh.tjmu.edu.cn

**This file includes:**

Supplementary Methods

References

Supplementary Figure 1 to 20

Supplementary Table 1 to 16

**Supplementary Methods**

**Plasma collection and RNA isolation**

Whole blood (5 mL) was collected into EDTA-containing tubes and separated into plasma and cellular fractions by centrifugation at 1000 g for 10 min. The cel-miR-39-3p mimics were added to each plasma sample (250 µL) as an exogenous control. Total plasma RNA was harvested with the TRIzol LS Reagent (Cat# 10296010, Thermo Fisher scientific, Shanghai, China) as described previously^1^. RNA was isolated from frozen heart tissues or cultured cells with TRIzol Reagent (Cat# 15596018, Thermo Fisher Scientific, Shanghai, China) according to the manufacturer’s protocol.

**Real-time quantitative reverse-transcription polymerase chain reaction (RT-PCR)**

Total RNA isolated from the plasma samples, heart tissues, or cells, was reversely transcribed using a first-strand cDNA synthesis kit (Cat# K1612, Thermo Fisher Scientific, Shanghai, China). The primers and Maxima SYBR Green/ROX qPCR Master Mix (Cat# K0231, Thermo Fisher Scientific, Shanghai, China) were used for real-time PCR to detect the relative expression of miRNA with the 7900HT Fast RealTime PCR system (Applied Biosystems, Foster City, CA) according to the manufacturer’s protocol. GAPDH was used as an endogenous control for mRNA. U6 was used as an endogenous control for miRNA in the cells or tissues. The raw Ct values were normalized by GAPDH or U6, and the 2^-ΔΔCt^ method was used in the subsequent analyses. In cellular experiments, the level of kshv-miR-K12-1-5p was shown directly using Ct values as previously reported, in which the sample contains a higher initial concentration of nucleic acid has a lower Ct value^2,3^.

For the absolute quantification of kshv-miR-K12-1-5p in plasma, a standard curve of the Ct values was obtained from serial dilutions (10^2^ to 10^7^ copies/μL) of the miRNA standard RNA (RiboBio, Guangzhou, China)^4^. The lower limit of quantification was 10^2^ copies/µL and the amplification efficiency was approximately 103%, as the correlation coefficient (R square) of the standard curve was 0.989. The Ct values from the plasma samples were plotted on the standard curves, and the kshv-miR-K12-1-5p copies/mL plasma was calculated. The samples below the lower limit of the quantification were imputed as 0. The RT-PCR for miRNAs was using a specific looped RT primer, combined with a specific forward primer and a universal reverse primer. These commercial primers for kshv-miR-K12-1-5p, U6 and miR-320a were designed and synthesized by from RiboBio (Cat# MQPS0002336, MQPS0000002 and MQPS0001042, RiboBio, Guangzhou, China). The other primers of mRNAs used are listed in Supplementary Table 15.

**Genetic DCM identification**

To identify the genetic DCM, we performed whole exome sequencing on genomic DNA of DCM patients and analyzed pathogenic variants on the 37 known causal genes for DCM. Pathogenic variants were defined as (1) Truncating variants (stop-gain, essential splice site and frameshift indel) and (2) rare missense variants with a Combined Annotation Dependent Depletion (CADD) score ≥ 20^5^. The whole exome sequencing was conducted by iGeneTech (Zhejiang, China).

**Propensity score-matched analysis**

To minimize baseline differences between DCM and non-DCM groups, we performed propensity score-matched analysis^6^, and variables included age, gender, smoking, history of hypertension, diabetes and hyperlipidemia. DCM and non-DCM groups were paired at 1:1 according to the propensity scores using nearest matching with a caliper size of 0.03.

**Enzyme-linked immunosorbent assay (ELISA)**

Tests for anti-KSHV IgG antibody were performed with a commercially available ELISA kit (Cat# 15-501-000, Advanced Biotechnologies Inc, MD) according to the manufacturer’s instructions. IFNβ levels in human or mouse plasma was measured using QuantiCyto® Human IFNβ ELISA kit (Cat# EHC026b, Neobioscience Technology Co., Ltd. Shenzhen, China) or QuantiCyto® Mouse IFNβ ELISA kit (Cat# EMC016, Neobioscience Technology Co,. Ltd. Shenzhen, China) according to the manufacturer’s instructions. TNF-α levels were measured using QuantiCyto® Mouse TNF-α ELISA kit (Cat# EMC102a, Neobioscience Technology Co., Ltd. Shenzhen, China).

**Immunohistochemical staining**

Human heart tissues fixed in formalin were paraffin embedded and cut into 4-µm-thick sections. For immunofluorescence, the sections were double-stained with anti-KSHV ORF73 or ORF45 and anti-cTNT (for cardiomyocytes), anti-CD31 (for endothelial cells), anti-Col1a1 (for fibroblasts) antibodies or WGA, respectively, following a previously described procedure^7^. For fluorescence staining in situ hybridization (FISH), the sections were stained using the probe of kshv-miR-K12-1-5p (RiboBio, Guangzhou, China) and cardiac cell markers’ antibodies following a previously described procedure^8^. Antibodies used are listed in Supplementary Table 16.

**Cell culture, infection and treatment**

Human cardiac microvascular endothelial cells (HCMECs) were obtained from ScienCell Research Laboratories (San Diego, CA) and cultured in Endothelial Cell Medium (Cat# 1001, ScienCell Research Laboratories, CA). AC16 cells were from the American Type Culture Collection (ATCC) and cultured in DMEM supplemented with 10% FBS. Cells were grown at 37 °C with an atmosphere of 95% air and 5% CO_2_. KSHV-positive cell line (BCBL-1) was maintained as described previously^9^. KSHV was produced and infected HCMECs as described previously^10^. The exosome was isolated from culture media of HCMECs with or without KSHV infection using Ribo^TM^ Exosome Isolation Reagent (Cat# C10130, RiboBio, Guangzhou, China). The protein and miRNA levels in the exosome were determined by Western blots and RT-PCR.

AC16 cells were transfected with kshv-miR-K12-1-5p mimics (100 nM, similarly hereinafter) or negative control (miR-con, 100 nM), respectively, using Lipo 2000 reagent according the manufacturer’s protocol. Then, cells were treated with IFNβ (100 U/ml) and incubated for 24 hours. Cells were lysed and measured by Western blots. After transfection, AC16 cells were infected with CVB3 at a MOI of 1 followed by IFNβ (100 U/ml) treatment. Twenty-four hours after infection, virus RNA level in cells were determined by RT-PCR. Cell apoptosis was evaluated by Annexin V-FITC/PI (BD Biosciences, Sparks, MD) and cell viability was evaluated by Cell Counting Kit-8 (HY-K0301, MedChemExpress, Shanghai, China).

For co-culture assay, AC16 cells were grown in the bottom well, while HCMECs or KSHV infected HCMECs were laid on the upper culture chamber of the Transwell co-culture plate (Cat# CLS3395, Merck KGaA, Darmstadt, Germany). Twenty-four hours after co-culture, AC16 cells were transfected with kshv-miR-K12-1-5p inhibitor (100 nM) and infected with CVB3 at a MOI of 1 followed by IFNβ (100 U/ml) treatment. Twenty-four hours after infection, virus RNA level in cells were determined by RT-PCR. Cell apoptosis and viability were evaluated as described earlier.

Human induced pluripotent stem cell-derived cardiomyocyte (hiPSC-derived CM) was obtained from Help Stem Cell Innovations (Nanjing, China). Cells were transfected with kshv-miR-K12-1-5p mimics (100 nM) and infected with CVB3 at a MOI of 0.5 followed by IFNβ (100 U/ml) treatment. Twelve hours after infection, virus RNA level in cells were determined by RT-PCR. Cell apoptosis was evaluated by TdT-mediated dUTP nick-end labeling (TUNEL) assay using in situ cell death detection kits (Roche Diagnostics GmbH, Mannheim, Germany) according to the manufacturer’s instructions. The detection of hiPSC-derived CM contractility was conducted as described previously^11^.

**Gene ontology analyses**

Using a cutoff of fold change > 2 and p < 0.05 (unadjusted p), 67 genes upregulated in Ago2-RIP seq following kshv-miR-K12-1-5p transfection compared with miR-con were used as gene sets for analysis. Gene ontology analyses were performed using WebGestalt (<http://www.webgestalt.org/option.php>)^12^.

**miRNA binding sites prediction**

The sequences of kshv-miR-K12-1-5p (MIMAT0002182, miRBase) and candidate genes were inputted and predicted in RNAhybrid^13^ (<https://bibiserv.cebitec.uni-bielefeld.de/rnahybrid/>). A minimum free energy (MFE) lower than -20 Kcal/mol was set as the filtering criterion.

**Bio-miR pulldown assay**

Twenty-four hours after transfection with biotin-labelled kshv-miR-K12-1-5p or miR-con (RiboBio, Guangzhou, China), AC16 cells were lysed and immunoprecipitation was performed using Dynabeads^TM^ M-280 Streptavidin beads (Cat# 60210, Thermo Scientific, Shanghai, China), as described previously^14^.

**Western blots**

Proteins from cell lysates (20 µg) were separated by 10% (wt/vol) SDS-polyacrylamide gel electrophoresis and transferred to PVDF membranes. After incubation with primary and secondary antibodies, the bands were visualized by enhanced chemiluminescence according to the manufacturer’s instructions (Thermo Scientific, Shanghai, China). The intensities of individual bands were analyzed by densitometry using ImageJ (National Institutes of Health Software, Bethesda, MD) and normalized to GAPDH level. Antibodies used are listed in Supplementary Table 16.

**Reporter constructs and luciferase reporter assays**

About 400 base pairs sequence containing the potential binding sites in 3′- untranslated region (UTR) of OAS2, IFIT1, IFIT3 or RSAD2 was synthesized and cloned into pMIR luciferase reporter vector (Cat# AM5795, Thermo Scientific, Shanghai, China), respectively. The full-length 5′-UTR of MX1 or IRF7 was synthesized and cloned into pGL3-Promoter vector (Cat# E176A, Promega, Beijing, China), respectively. For reporter assays, HEK293 cells were plated in 24-well plates and co-transfected kshv-miR-K12-1-5p mimics or control with 250 ng/well of reporter and TK plasmid. Luciferase activity was measured 24 hours after transfections with the Dual-Luciferase® Reporter Assay System (Cat# E1910, Promega, Beijing, China).

**Echocardiography and in vivo hemodynamics**

After mice were anesthetized with 1.5% isoflurane, echocardiographic measurements were performed using a high-resolution imaging system with a 30-MHz high-frequency scanhead (Vevo770, VisualSonics Inc, Toronto, Canada) as described previously^15^. After mice were anesthetized with intraperitoneal injection of the mixture of ketamine and xylazine, the in vivo hemodynamic analyses were performed using a Millar Catheter System (Millar 1.4F, SPR 835, Millar Instruments Inc, Houston, TX) as described previously^15^.

**Histology staining**

Heart tissues fixed in formalin were paraffin embedded and cut into 4-µm-thick sections and stained with hematoxylin-eosin (H&E) and Sirius red. The inflammation histoscore was evaluated in a blind manner by an investigator who used light microscopy, according to a scoring system from 0 to 4 (0, no inflammatory infiltrates; 1, small foci of inflammatory cells between myocytes; 2, larger foci of > 100 inflammatory cells; 3, > 10% of a cross-section involved; and 4, > 30% of a cross-section involved) as described previously^16^.

**Transmission electron microscope (TEM)**

A portion of each heart sample was immediately fixed by immersion in 3% glutaraldehyde, 2% paraformaldehyde, and 0.1 M cacodylate buffer (pH 7.4) containing 0.1 M sucrose and 3 mM CaCl_2_ for 30 min. The samples were then cut into 1-mm^3^ blocks, which was followed by fixation at 4 °C for 24 h in the same solution. Heart blocks were fixed in 1% osmium tetroxide for 2 h and embedded in epoxy resin (Araldite; Sigma-Aldrich), and then cut into 6-μm sections using a microtome. Images were acquired using a transmission electron microscope (JEM-1010, JEOL, Japan).

**Atomic force microscopy (AFM)**

Heart tissues fixed in formalin were paraffin embedded and cut into 4-µm-thick sections. After dewaxing and hydration, the sections were observed using microscope (JSPM-4210, JEOL, Japan) and the roughness of the surface was calculated^17^.

**Sarcomere shortening measurements**

Adult cardiac myocytes were isolated from adult male BALB/c mice and the sarcomere shortening measurements were performed with a protocol worked out based on previous studies^11^.

**References**

1 Long, G. *et al.* Circulating miR-30a, miR-126 and let-7b as biomarker for ischemic stroke in humans. *BMC. Neurol.* **13**, 178 (2013).

2 McNamara, R. P. *et al.* Extracellular vesicles from Kaposi Sarcoma-associated herpesvirus lymphoma induce long-term endothelial cell reprogramming. *PLoS. Pathog.* **15**, e1007536 (2019).

3 Green, M. R. & Sambrook, J. Analysis and Normalization of Real-Time Polymerase Chain Reaction (PCR) Experimental Data. *Cold. Spring. Harb. Protoc.* **2018** (2018).

4 Iguchi, T., Niino, N., Tamai, S., Sakurai, K. & Mori, K. Absolute Quantification of Plasma MicroRNA Levels in Cynomolgus Monkeys, Using Quantitative Real-time Reverse Transcription PCR. *J. Vis. Exp.* (2018).

5 Kircher, M. *et al.* A general framework for estimating the relative pathogenicity of human genetic variants. *Nat. Genet.* **46**, 310-315 (2014).

6 Cui, H. *et al.* Untargeted metabolomics identifies succinate as a biomarker and therapeutic target in aortic aneurysm and dissection. *Eur. Heart. J.* **42**, 4373-4385 (2021).

7 Zhao, Y. *et al.* MiR-30c protects diabetic nephropathy by suppressing epithelial-to-mesenchymal transition in db/db mice. *Aging Cell* **16**, 387-400 (2017).

8 de Planell-Saguer, M., Rodicio, M. C. & Mourelatos, Z. Rapid in situ codetection of noncoding RNAs and proteins in cells and formalin-fixed paraffin-embedded tissue sections without protease treatment. *Nat. Protoc.* **5**, 1061-1073 (2010).

9 Renne, R. *et al.* Lytic growth of Kaposi's sarcoma-associated herpesvirus (human herpesvirus 8) in culture. *Nat. Med.* **2**, 342-346 (1996).

10 Bridge, G. *et al.* The microRNA-30 family targets DLL4 to modulate endothelial cell behavior during angiogenesis. *Blood* **120**, 5063-5072 (2012).

11 Fan, J. *et al.* LncRNA ZNF593-AS Alleviates Contractile Dysfunction in Dilated Cardiomyopathy. *Circ. Res.* **128**, 1708-1723 (2021).

12 Liao, Y., Wang, J., Jaehnig, E. J., Shi, Z. & Zhang, B. WebGestalt 2019: gene set analysis toolkit with revamped UIs and APIs. *Nucleic Acids Res.* **47**, W199-W205 (2019).

13 Rehmsmeier, M., Steffen, P., Hochsmann, M. & Giegerich, R. Fast and effective prediction of microRNA/target duplexes. *RNA* **10**, 1507-1517 (2004).

14 Awan, H. M. *et al.* Comparing two approaches of miR-34a target identification, biotinylated-miRNA pulldown vs miRNA overexpression. *RNA Biol.* **15**, 55-61 (2018).

15 Chen, C. *et al.* Mir30c Is Involved in Diabetic Cardiomyopathy through Regulation of Cardiac Autophagy via BECN1. *Mol. Ther. Nucleic Acids* **7**, 127-139 (2017).

16 Eriksson, U. *et al.* Lethal autoimmune myocarditis in interferon-gamma receptor-deficient mice: enhanced disease severity by impaired inducible nitric oxide synthase induction. *Circulation* **103**, 18-21 (2001).

17 Picos, A. M. *et al.* Atomic force microscopy analysis of the surface alterations of enamel, dentin, composite and ceramic materials exposed to low oral pH in GERD. *Exp. Ther. Med.* **22**, 673 (2021).

**Supplementary Figures**

**Supplementary Figure 1.**

**
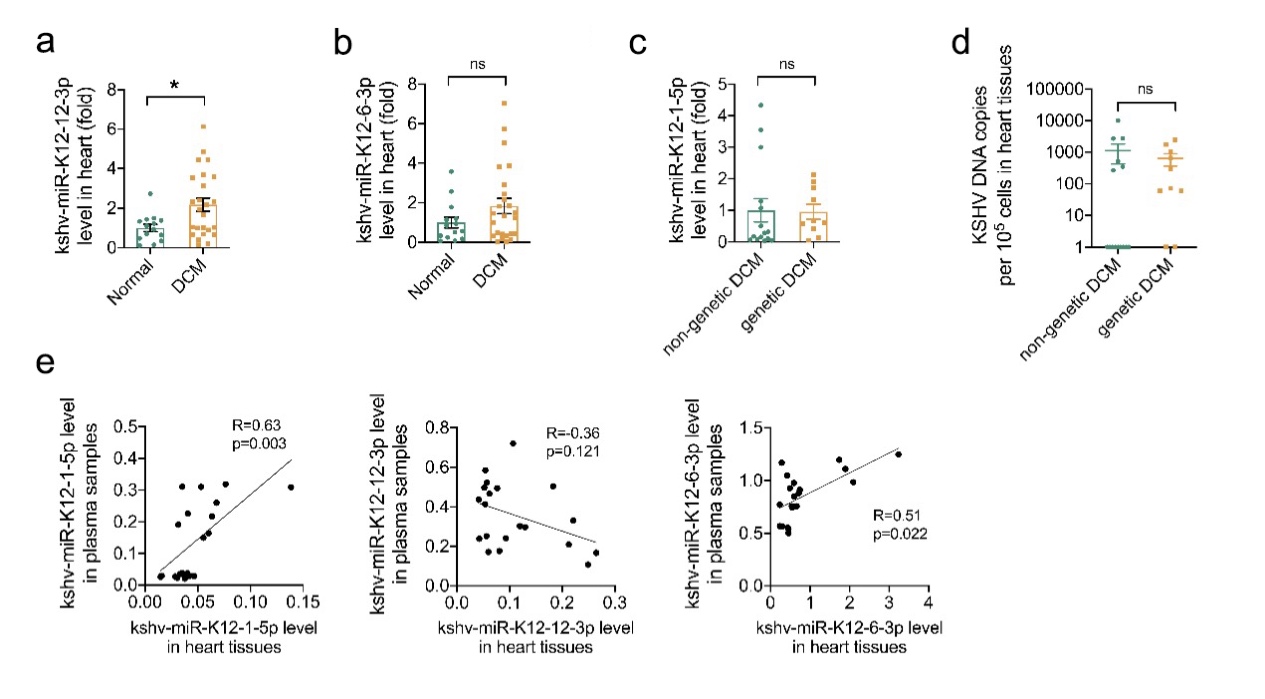
**

**Supplementary Figure 1.** The human cardiac level of kshv-miR-K12-1-5p was positively correlated with its level in plasma samples. Relative levels of kshv-miR-K12-12-3p (a) and kshv-miR-K12-6-3p (b) in the heart from normal (healthy donors, n=14) and DCM patients (n=25). (c) Relative levels of kshv-miR-K12-1-5p in the heart from non-genetic (n=15) and genetic DCM patients (n=10). (d) The KSHV DNA copies in the heart from non-genetic (n=15) and genetic DCM patients (n=10). (e) The correlation analyses between the cardiac and plasma levels of kshv-miR-K12-1-5p, kshv-miR-K12-12-3p or kshv-miR-K12-6-3p (miR profiles data). *p<0.05.

**Supplementary Figure 2.**


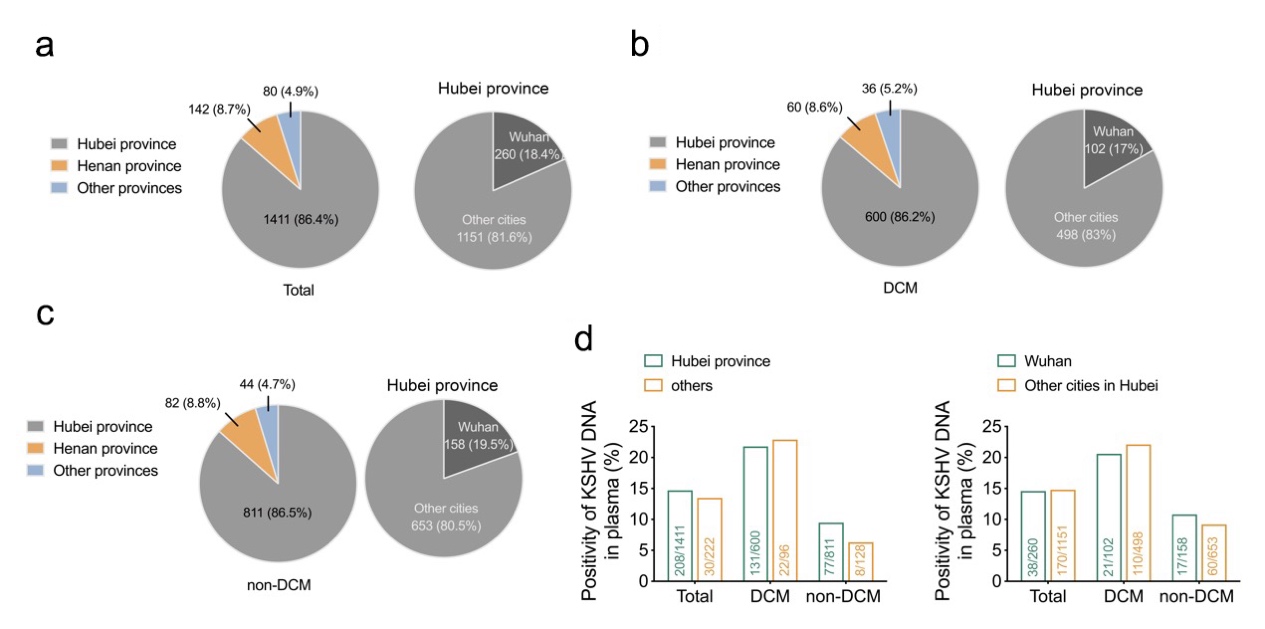


**Supplementary Figure 2.** Regional distribution of patients included in the plasma KSHV DNA and miRNA detection. Regional distribution of total (a), DCM (b) and non-DCM (c) populations. (d) The positivity of KSHV DNA in plasma of patients from Hubei and other provinces (left), as well as Wuhan and other cities in Hubei (right).

**Supplementary Figure 3.**


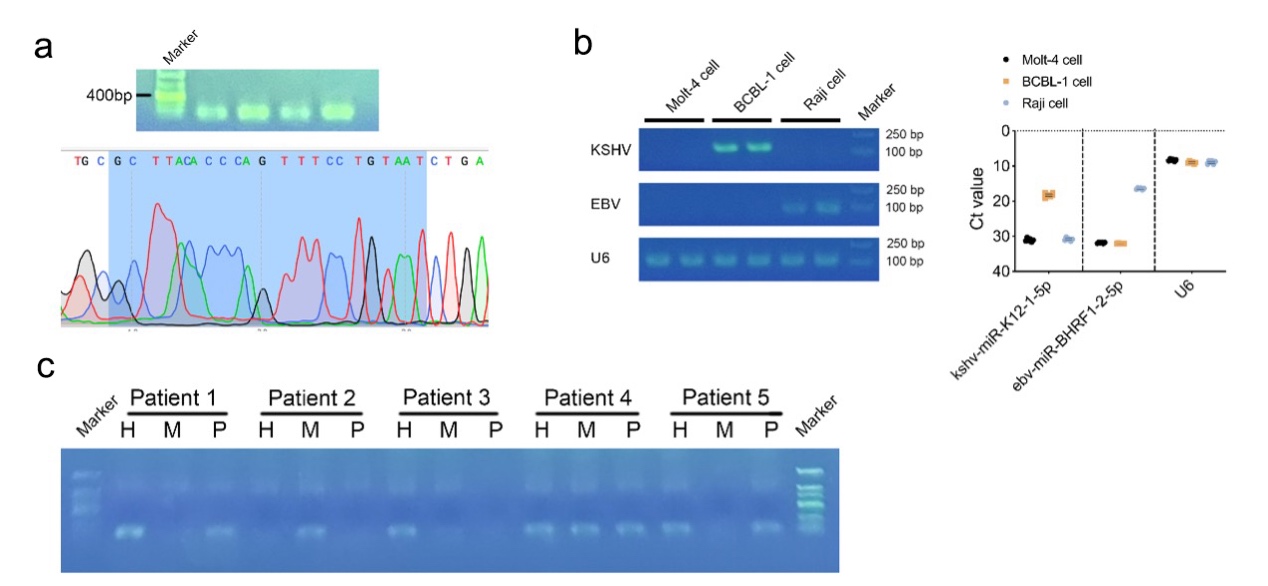


**Supplementary Figure 3.** The specificity and source of kshv-miR-K12-1-5p detected in plasma. (a) The agarose gel (up) and Sanger sequencing (down) of real-time PCR products of kshv-miR-K12-1-5p in plasma detection. (b) (left) The agarose gel of KSHV and EBV DNA detection in Molt-4 (neither KSHV nor EBV-infected), BCBL-1 (KSHV-infected) and Raji (EBV-infected) cell lines. (right) The levels of kshv-miR-K12-1-5p and ebv-miR-BHRF1-2-5p in these cell lines. (c) The agarose gel of KSHV DNA detection in the corresponding heart (H), peripheral blood mononuclear cell (M) and plasma (P) samples from 5 DCM patients.

**Supplementary Figure 4.**

**
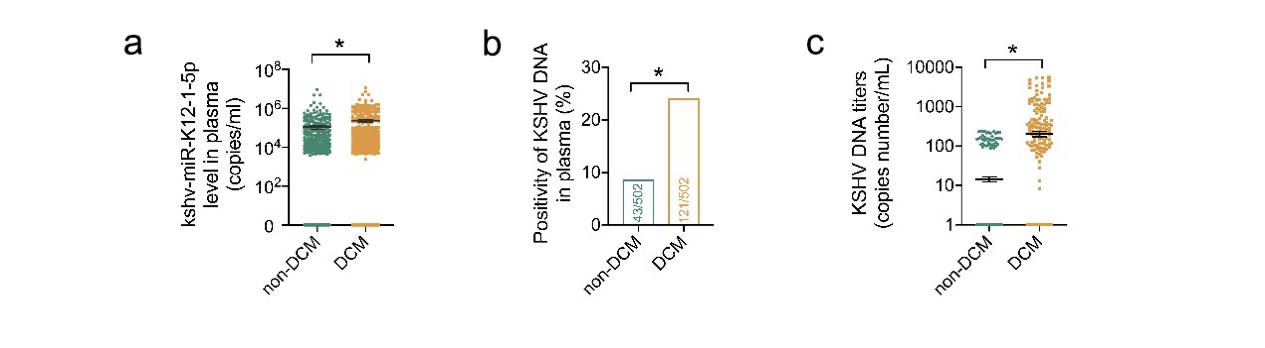
**

**Supplementary Figure 4.** The level of kshv-miR-K12-1-5p and KSHV DNA were increased in plasma samples from DCM patients compared to non-DCM after propensity score-matched analysis. 502 DCM cases were matched at a 1:1 ratio with 502 non-DCM subjects. The copies of kshv-miR-K12-1-5p (a), the positivity of KSHV DNA (b) and KSHV DNA copies (c) in the plasma samples were measured in two groups. *p<0.05.

**Supplementary Figure 5.**

**
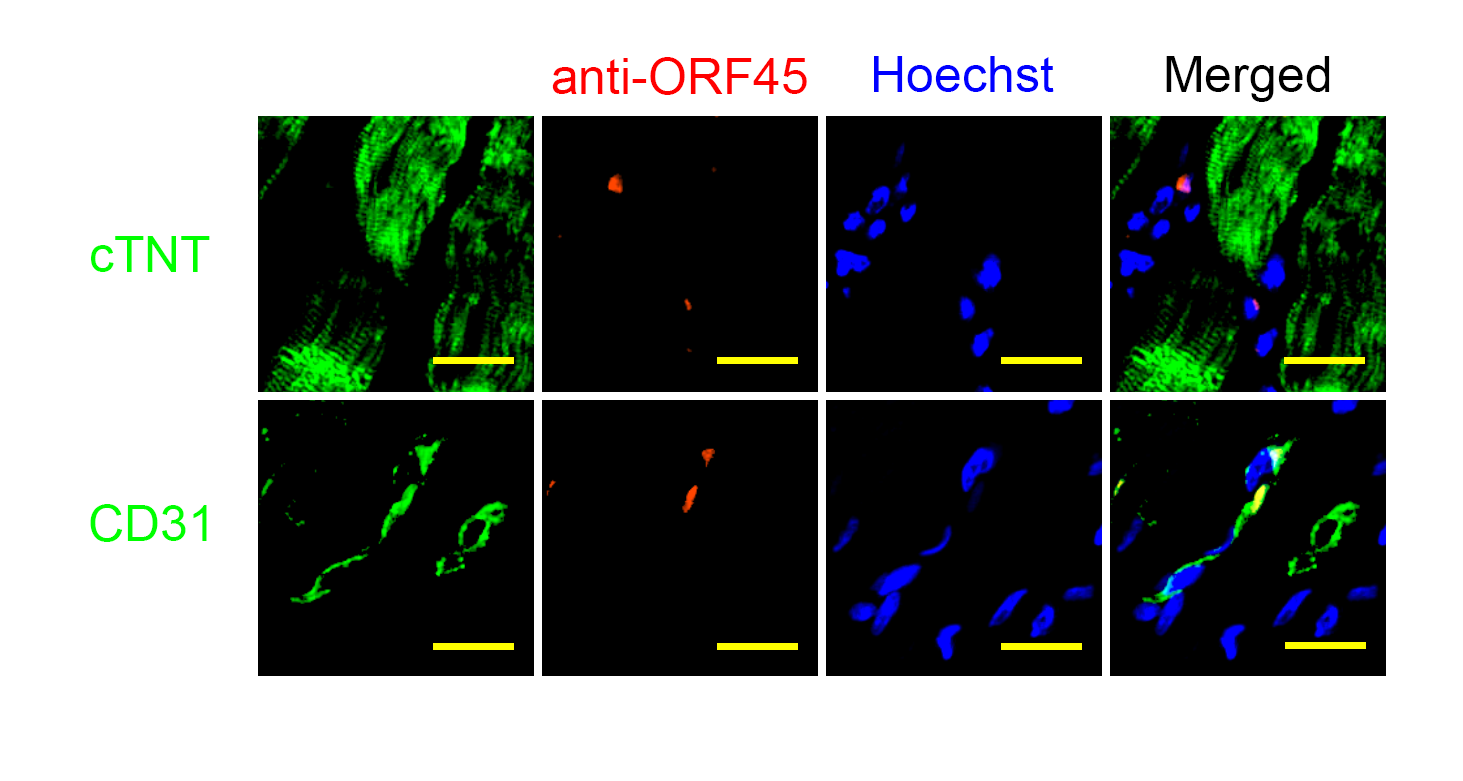
**

**Supplementary Figure 5.** Representative images of immunofluorescence staining of KSHV ORF45 (red) as well as markers of cardiomyocytes (cTNT) and endothelial cells (CD31). Scale bar=20µm.

**Supplementary Figure 6.**

**
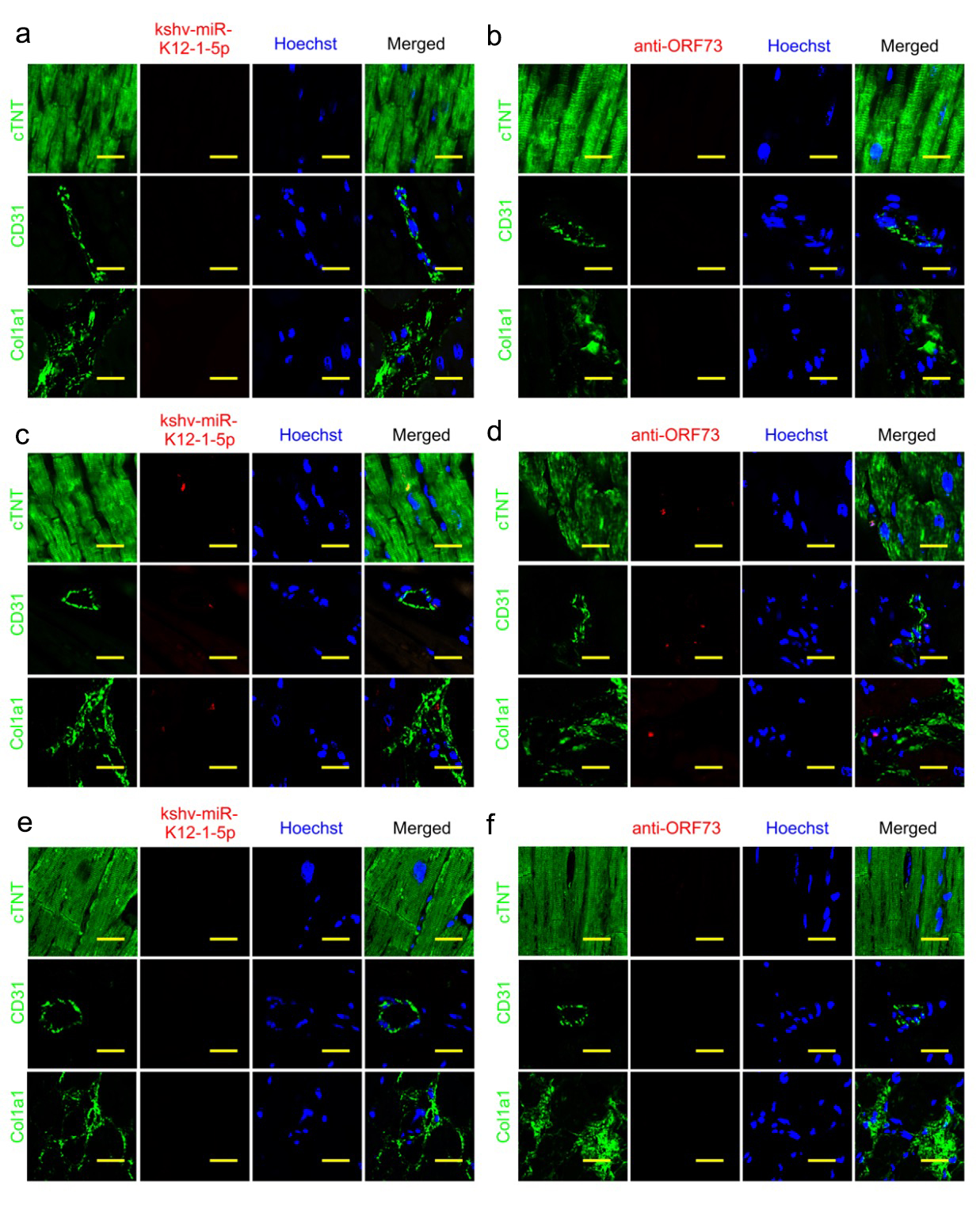
**

**Supplementary Figure 6.** Representative images of fluorescence in situ hybridization of kshv-miR-K12-1-5p (red) or immunofluorescence staining of KSHV ORF73 (red) as well as markers of cardiomyocytes (cTNT), endothelial cells (CD31) or fibroblasts (Col1a1) in normal heart with KSHV negativity (a-b), normal heart with KSHV positivity (c-d) and KSHV negative DCM heart (e-f). Scale bar=20µm.

**Supplementary Figure 7**

**
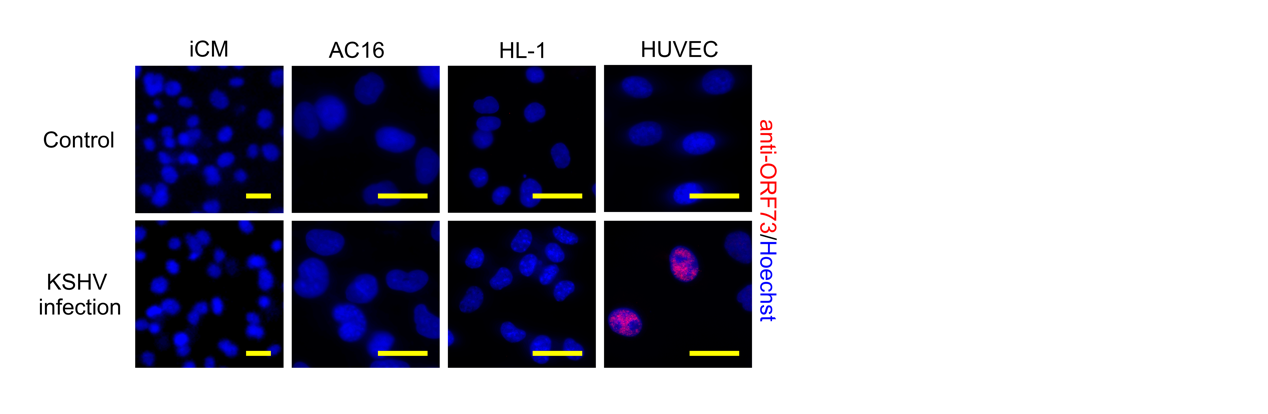
**

**Supplementary Figure 7.** Representative images of immunofluorescence staining of KSHV ORF73 (red) in KSHV-infected iCM (hiPSC-derived cardiomyocytes), AC16 (human cardiomyocyte cell line), HL-1 (mouse cardiomyocyte cell line) and HUVEC (human endothelial cell line). Scale bar=25µm.

**Supplementary Figure 8.**

**
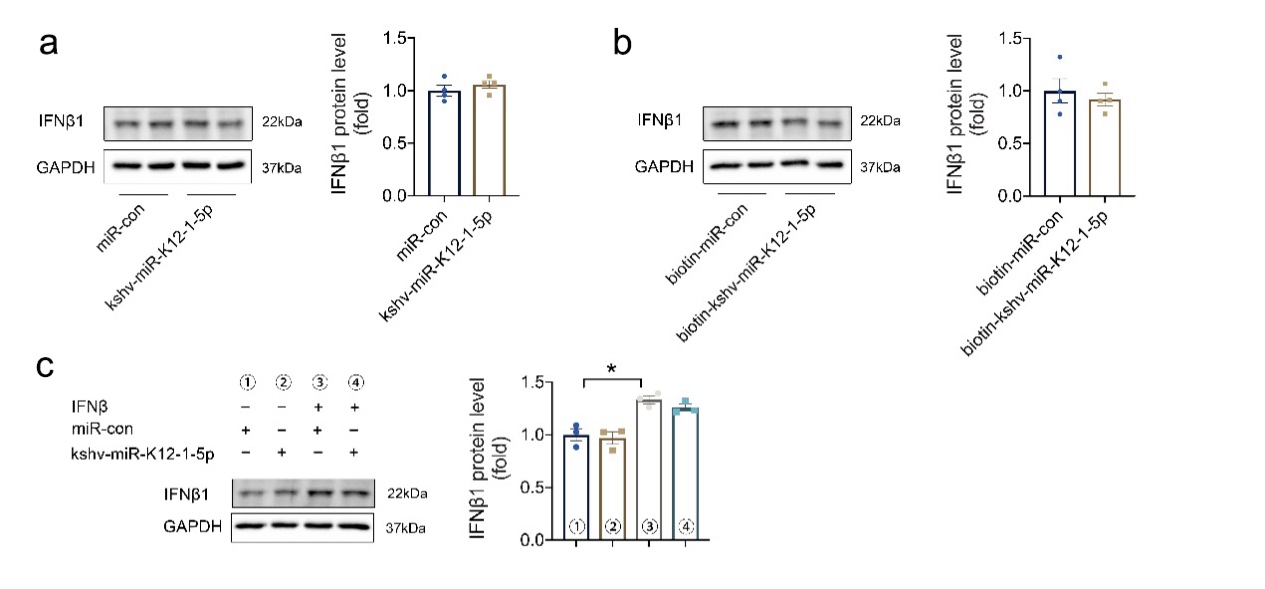
**

**Supplementary Figure 8.** kshv-miR-K12-1-5p had no effect on IFNβ1 expression in AC16 cells. (a) The expression of IFNβ1 detected by Western blot in AC16 cells with kshv-miR-K12-1-5p transfection (n=4). (b) The expression of IFNβ1 detected by Western blot in AC16 cells with biotin-labelled kshv-miR-K12-1-5p transfection (n=4). (c) The expression of IFNβ1 detected by Western blot in AC16 cells with kshv-miR-K12-1-5p transfection followed by IFNβ treatment (n=3). *p<0.05.

**Supplementary Figure 9.**


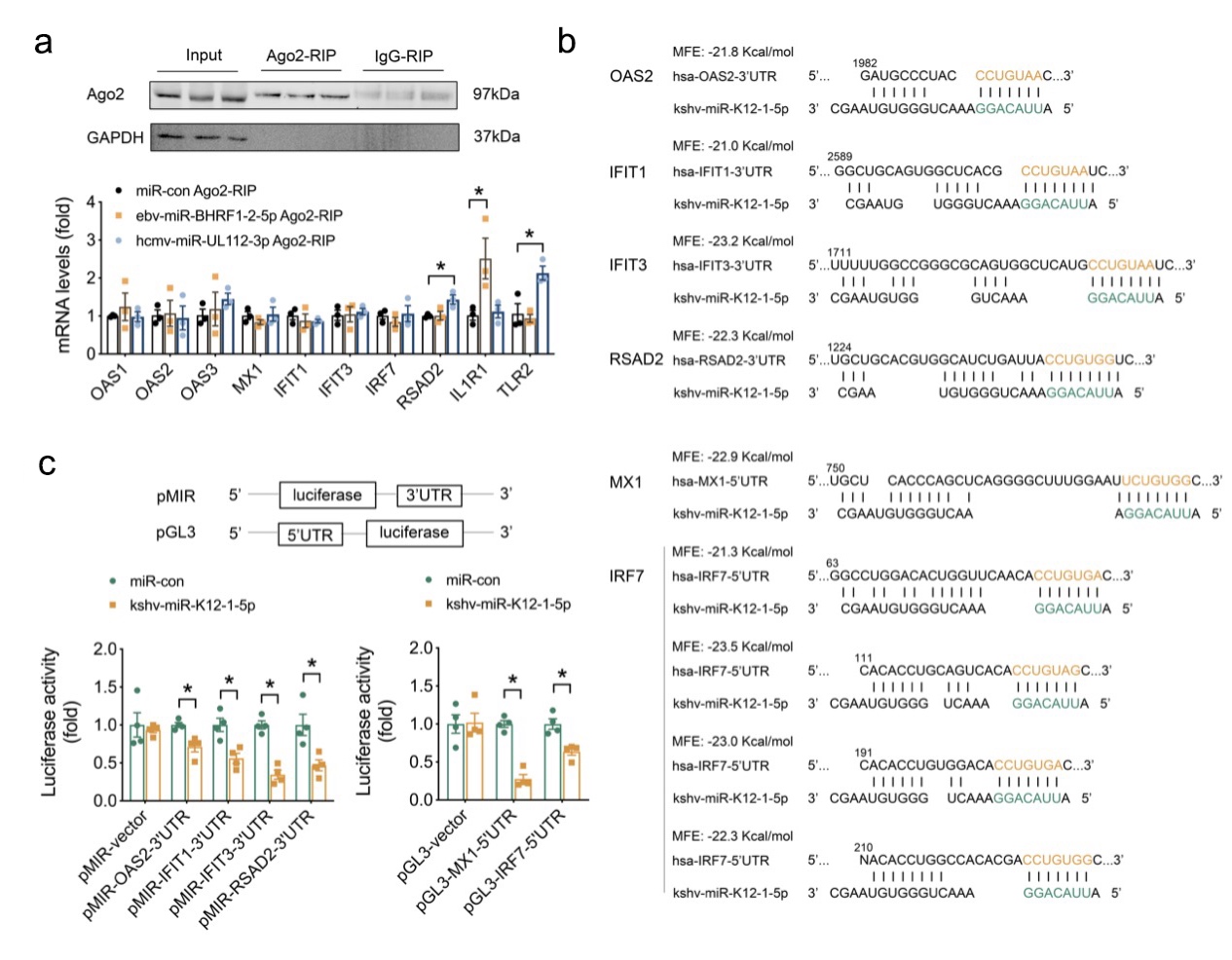


**Supplementary Figure 9.** Ago2-RIP and luciferase reporter assay of type I IFN genes. (a) The protein levels detected by Western blots (up) and RNA levels detected by RT-PCR (down) following Ago2-RIP (n=6 each). (b-c) The luciferase reporter containing the potential binding sites in 3’-UTR of human OAS2, IFIT1, IFIT3, or RSAD2, as well as in 5’-UTR of MX1 or IRF7, was co-transfected with kshv-miR-K12-1-5p mimics into HEK293 cells and luciferase activities were measured (n=4 each). *p<0.05.

**Supplementary Figure 10.**


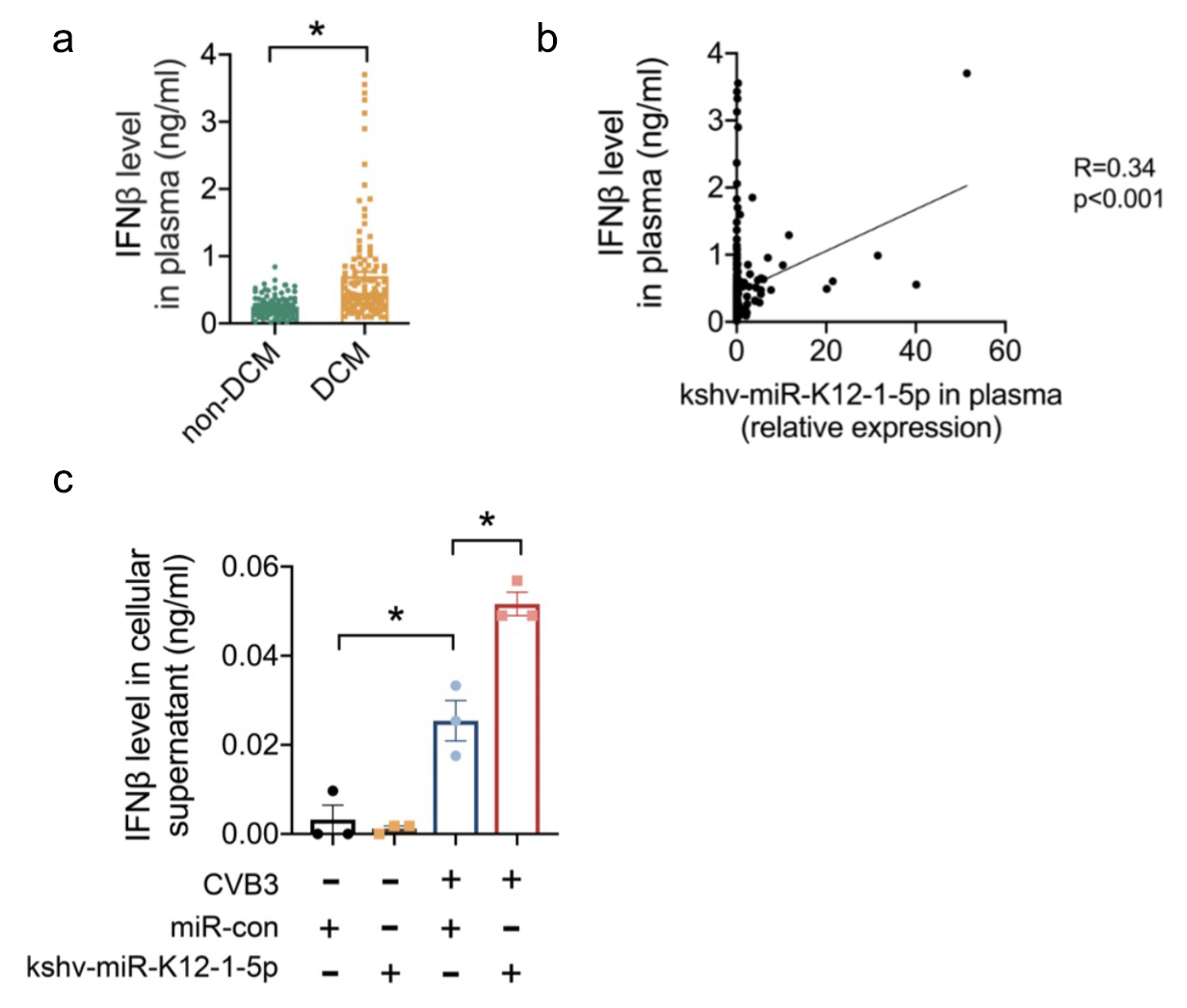


**Supplementary Figure 10.** The IFNβ level in plasma from patients and treated cells. (a) The IFNβ level in plasma from non-DCM patients (n=122) and DCM patients (n=123). (b) The correlation analyses between the IFNβ level and kshv-miR-K12-1-5p level in plasma. (c) The level of IFNβ in cellular supernatant of AC16 cells with kshv-miR-K12-1-5p transfection followed by CVB3 infection. *p<0.05.

**Supplementary Figure 11.**

**
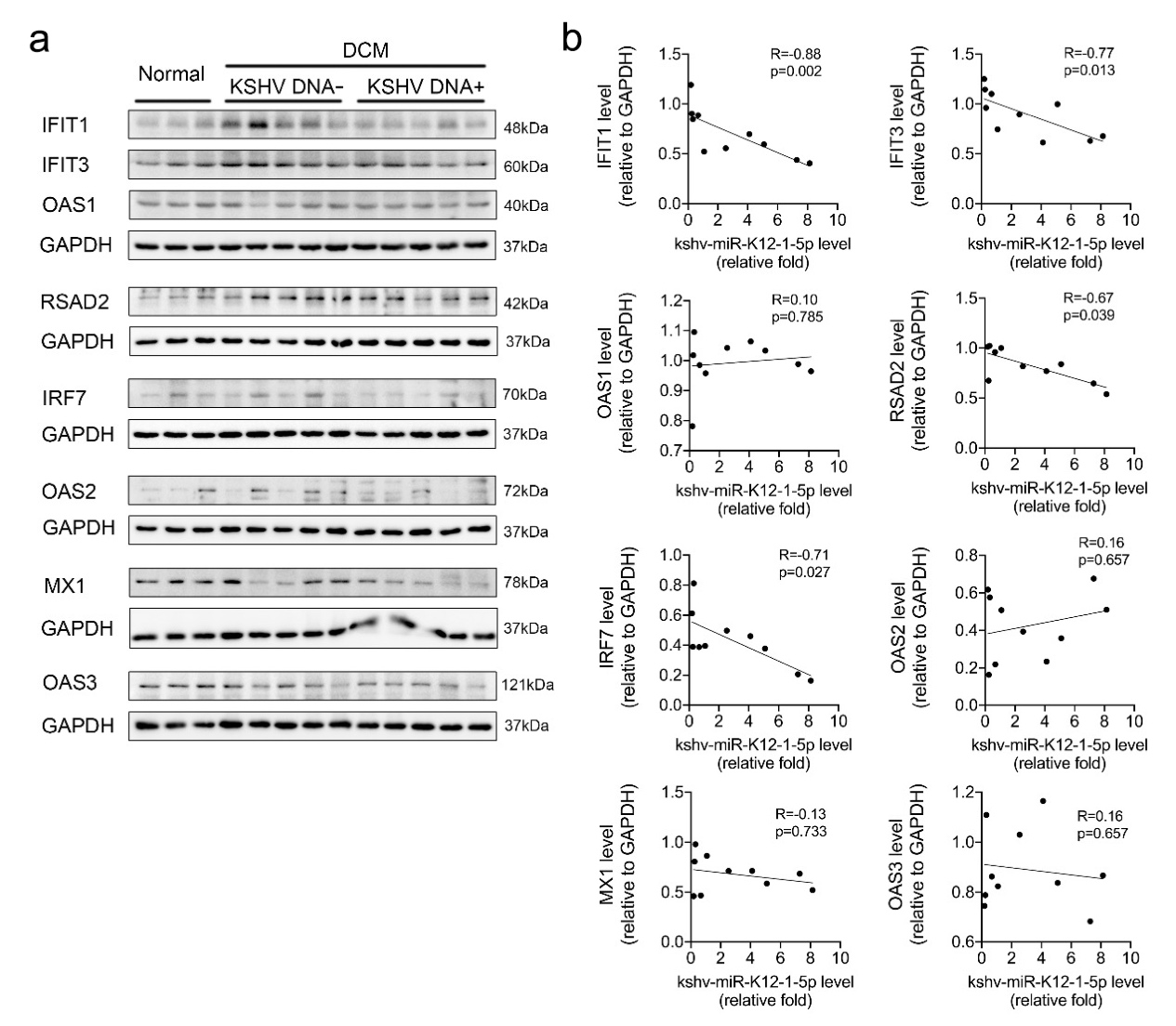
**

**Supplementary Figure 11.** The expression of IFNβ1 downstream signals was negatively correlated with kshv-miR-K12-1-5p level in DCM hearts. (a)The expression of IFNβ1 downstream signals detected by Western blot in human normal and DCM heart samples. (b) The correlation analyses between the IFNβ1 downstream signals expression and kshv-miR-K12-1-5p level in DCM heart samples.

**Supplementary Figure 12.**


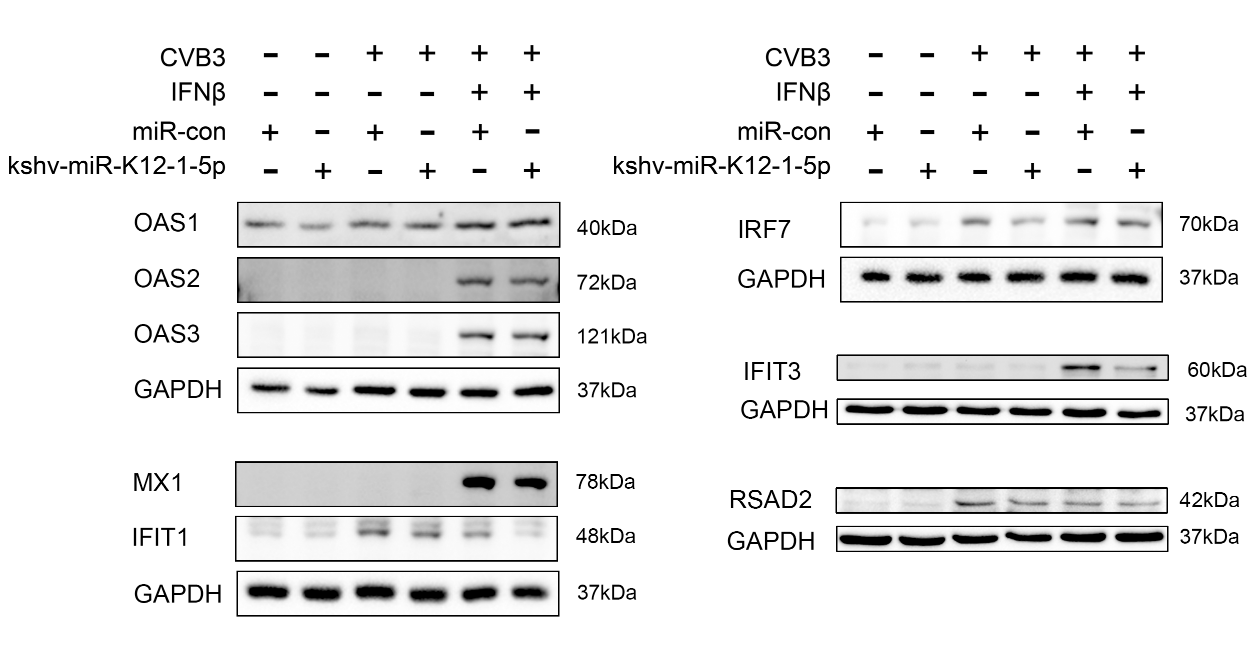


**Supplementary Figure 12.** The expression of candidate target genes detected by Western blot in AC16 cells with kshv-miR-K12-1-5p transfection followed by CVB3 infection and IFNβ treatment.

**Supplementary Figure 13.**


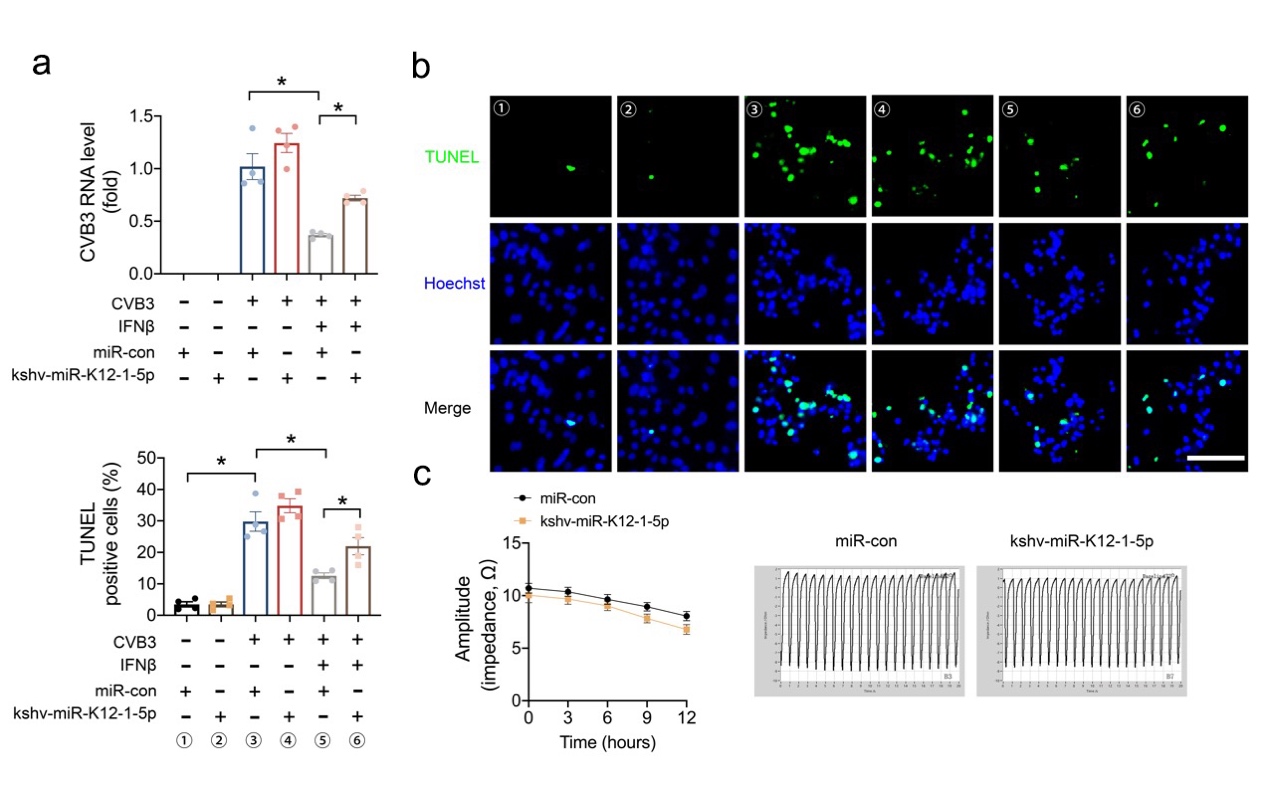


**Supplementary Figure 13.** kshv-miR-K12-1-5p weakened the anti-viral effects of IFNβ in hiPSC-derived CM. hiPSC-derived CM was transfected with kshv-miR-K12-1-5p mimics or miR-con, followed by CVB3 infection and IFNβ treatment. Twelve hours later, the CVB3 RNA level in cells was detected by RT-PCR (a) and the apoptotic cells were detected by TdT-mediated dUTP nick-end labeling (TUNEL) assay (b) (n=4 each). (c) Myocardial contractility of hiPSC-derived CM detected by patch-clamping (n=5 each). *p<0.05.

**Supplementary Figure 14.**


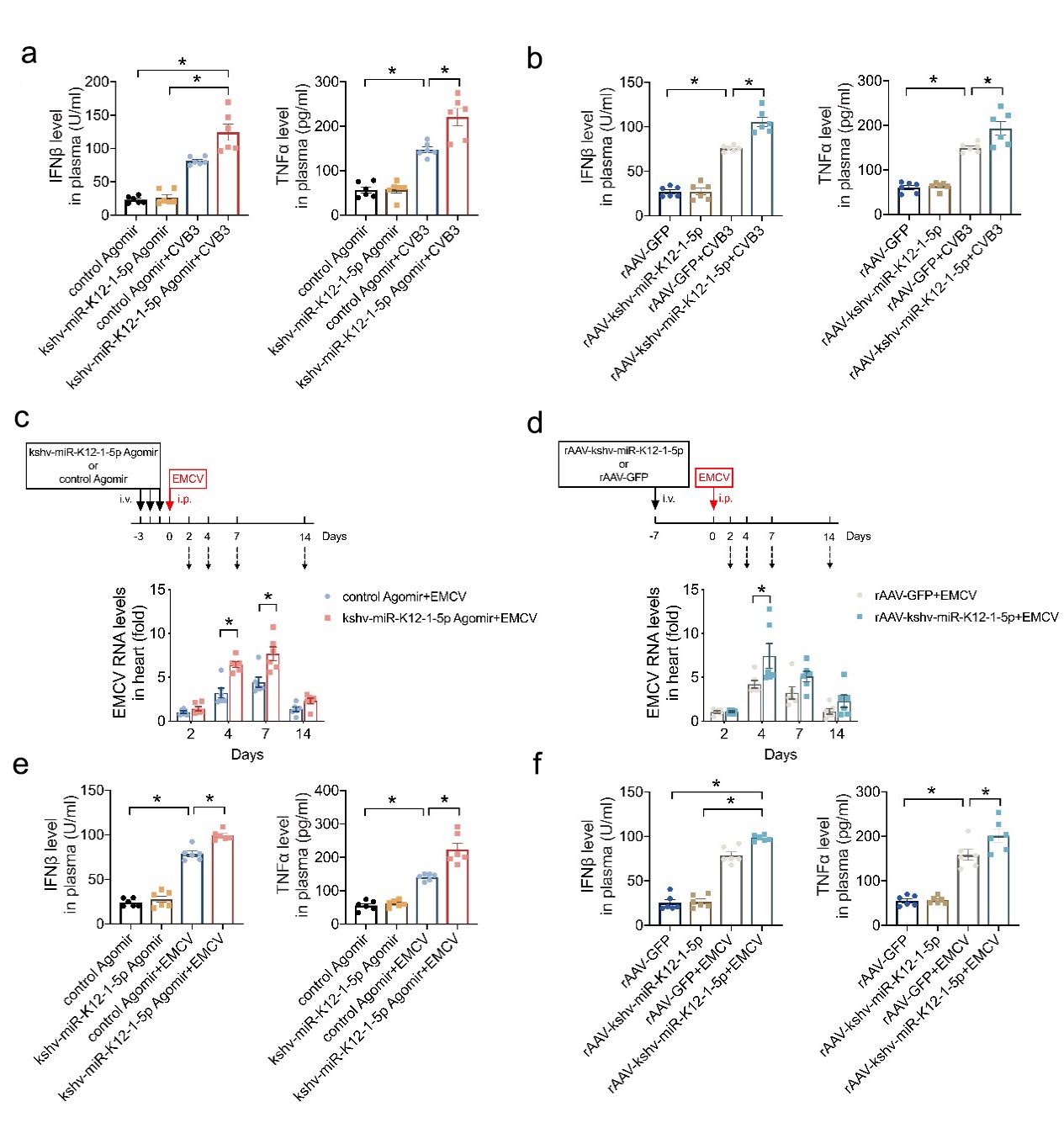


**Supplementary Figure 14.** kshv-miR-K12-1-5p increased serum inflammatory cytokines and viral replication post virus infection. (a) and (b) IFNβ and TNFα level in plasma detected by ELISA among different groups on day 7. (c) and (d) Schema of the experimental setup (up) and the EMCV RNA levels detected by RT-PCR in the heart tissues at different time points. i.v., tail vein injection; i.p., intraperitoneal injection. (e) and (f) IFNβ and TNFα levels in plasma detected by ELISA among different groups on day 7. n=6 each. *p<0.05.

**Supplementary Figure 15.**


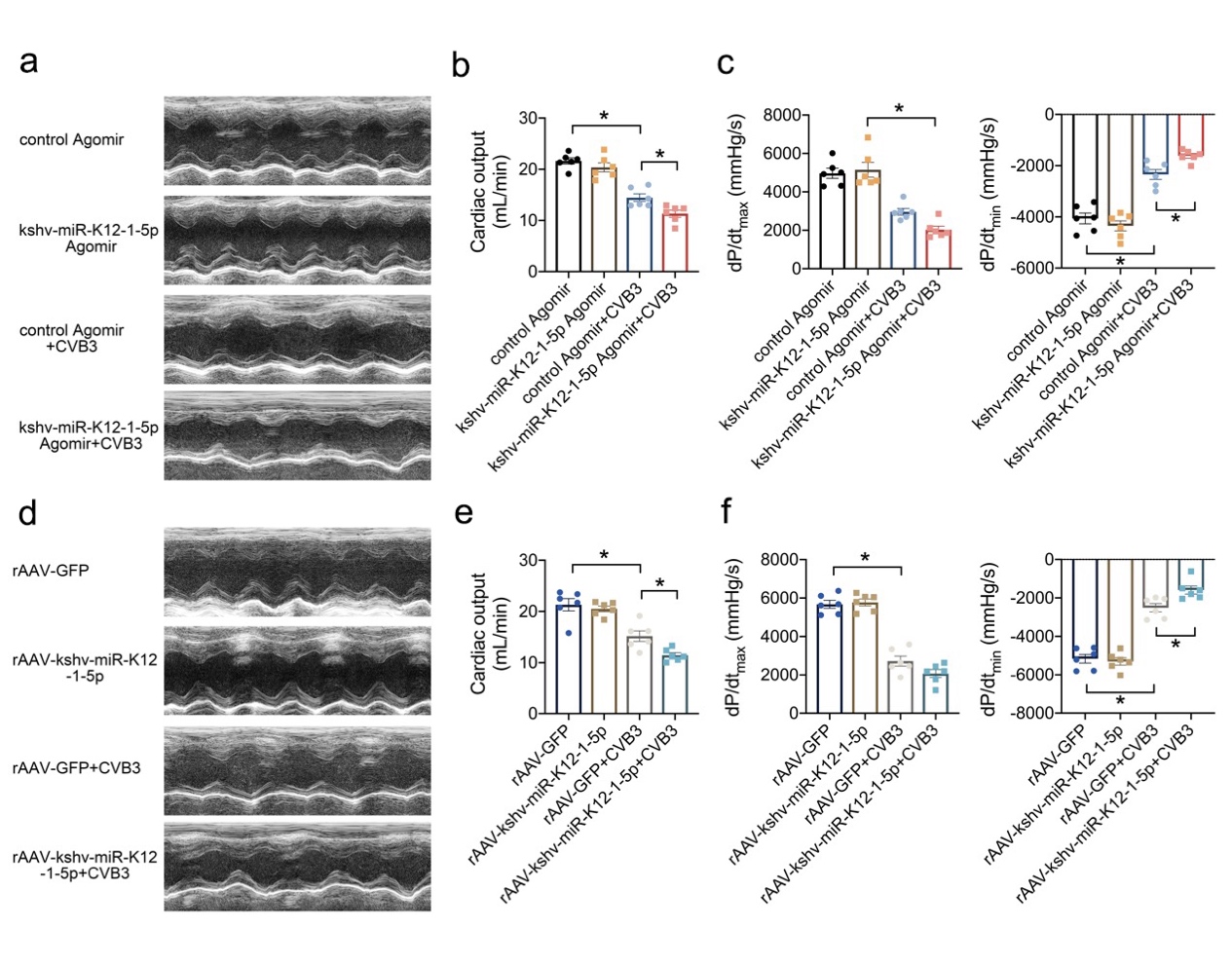


**Supplementary Figure 15.** kshv-miR-K12-1-5p aggravated cardiac output decrease and diastolic dysfunction induced by CVB3 infection at day 14. (a) and (d) Representative images of M-mode echocardiography in mice. (b) and (e) Cardiac output of mice with different treatments measured by echocardiography. (c) and (f) Cardiac function in mice measured by in vivo hemodynamics. dP/dt_max_, maximal left ventricular pressure rising rate; dP/dt_mjn_, minimal left ventricular pressure rising rate. n=6 each. *p<0.05.

**Supplementary Figure 16**

**
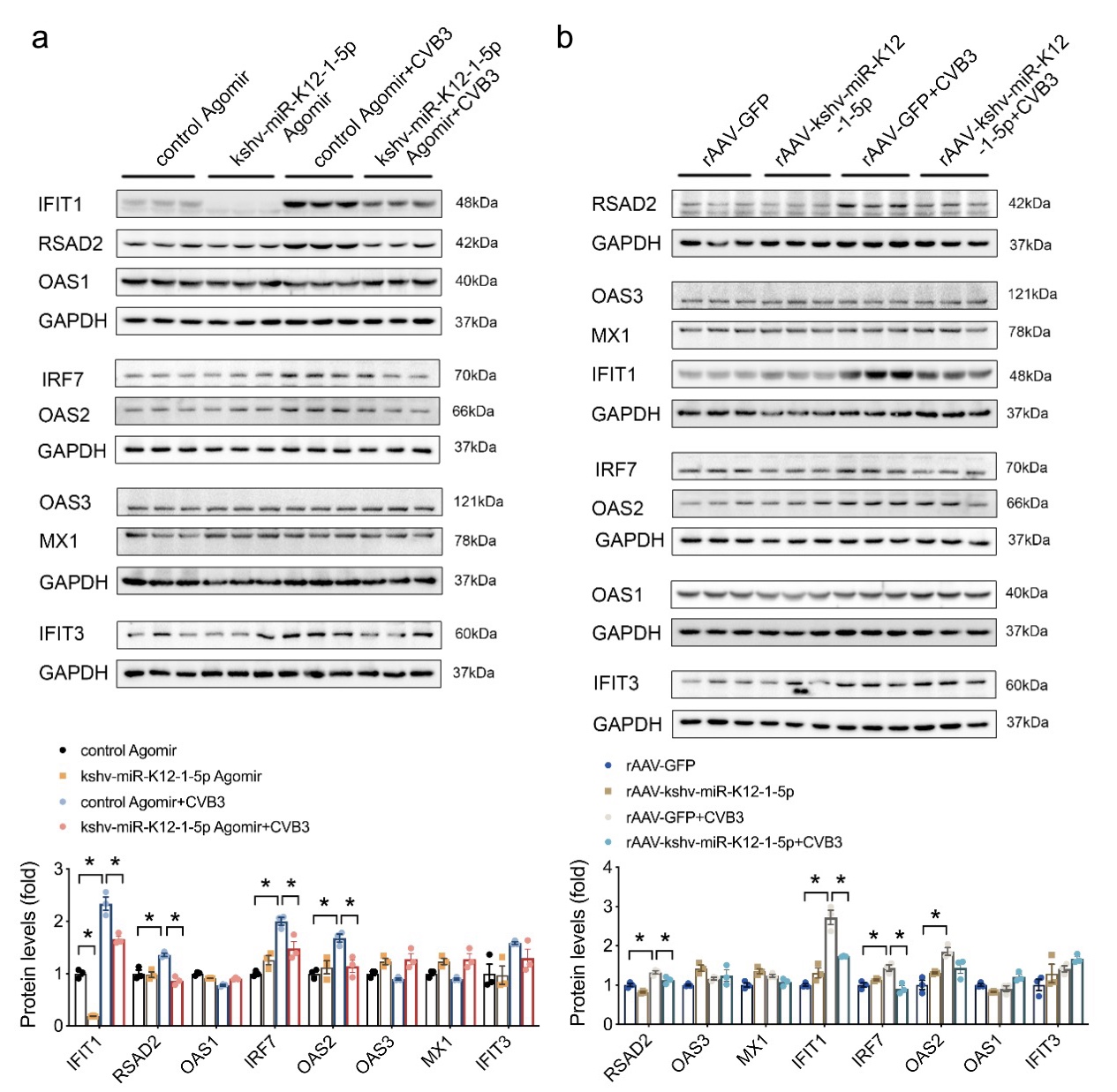
**

**Supplementary Figure 16.** The expression of IFNβ1 downstream genes was downregulated by kshv-miR-K12-1-5p in mice models. The expression of IFNβ1 downstream genes detected by Western blot in CVB3 infected mice with kshv-miR-K12-1-5p overexpression using agomiR (a) or rAAV (b). *p<0.05.

**Supplementary Figure 17.**


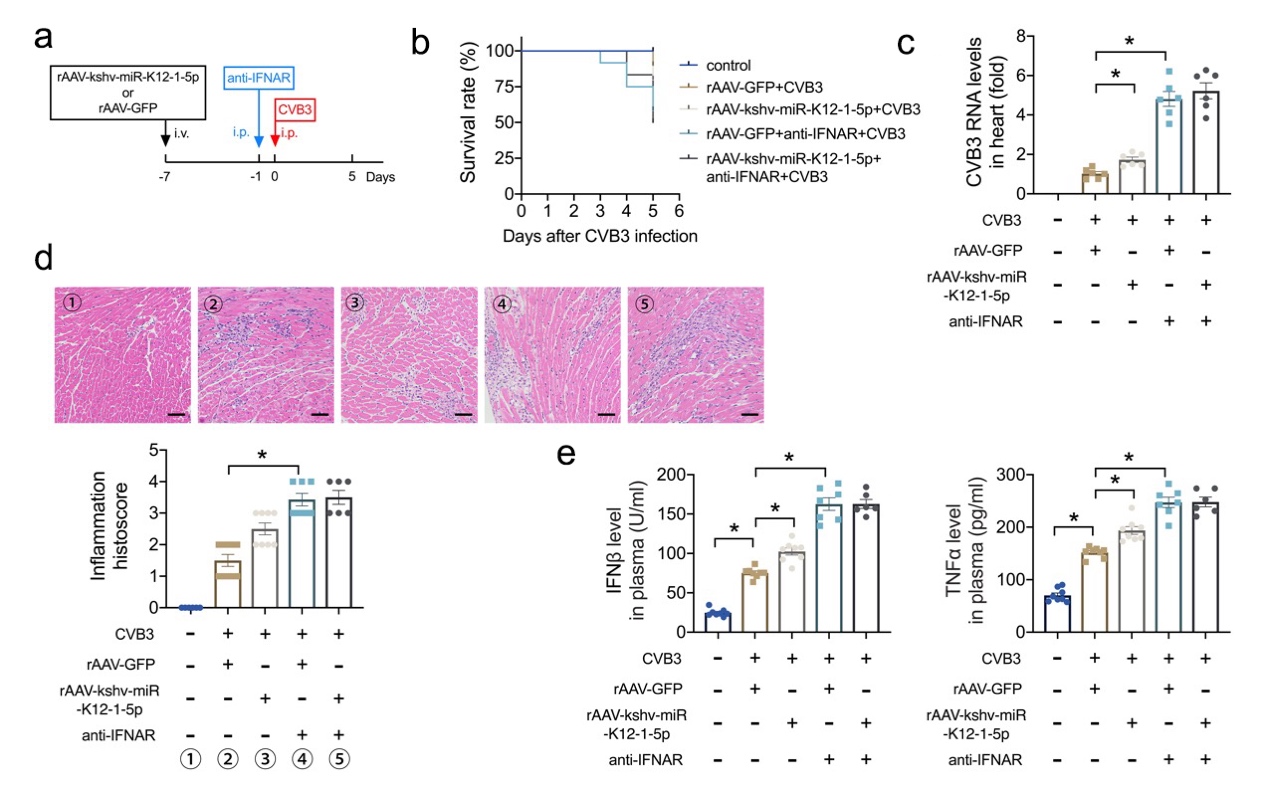


**Supplementary Figure 17.** kshv-miR-K12-1-5p could not aggravate CVB3 induced cardiac inflammatory infiltration after type I IFN signaling blocking. (a) Schema of the experimental setup for blocking type I IFN signaling via anti-IFNAR mAB. i.v., tail vein injection; i.p., intraperitoneal injection. (b) Survival of mice infected with CVB3 with and without prior administration of anti-IFNAR. (c) The CVB3 RNA levels detected by RT-PCR in the heart tissues on 5 days. (d) HE staining of representative heart tissue sections (up) and the inflammation histoscore calculated based on HE staining (down) on day 5. Scale bar=100µm. (e) IFNβ (left) and TNFα (right) level in plasma detected by ELISA among different groups on day 5. *p<0.05.

**Supplementary Figure 18.**


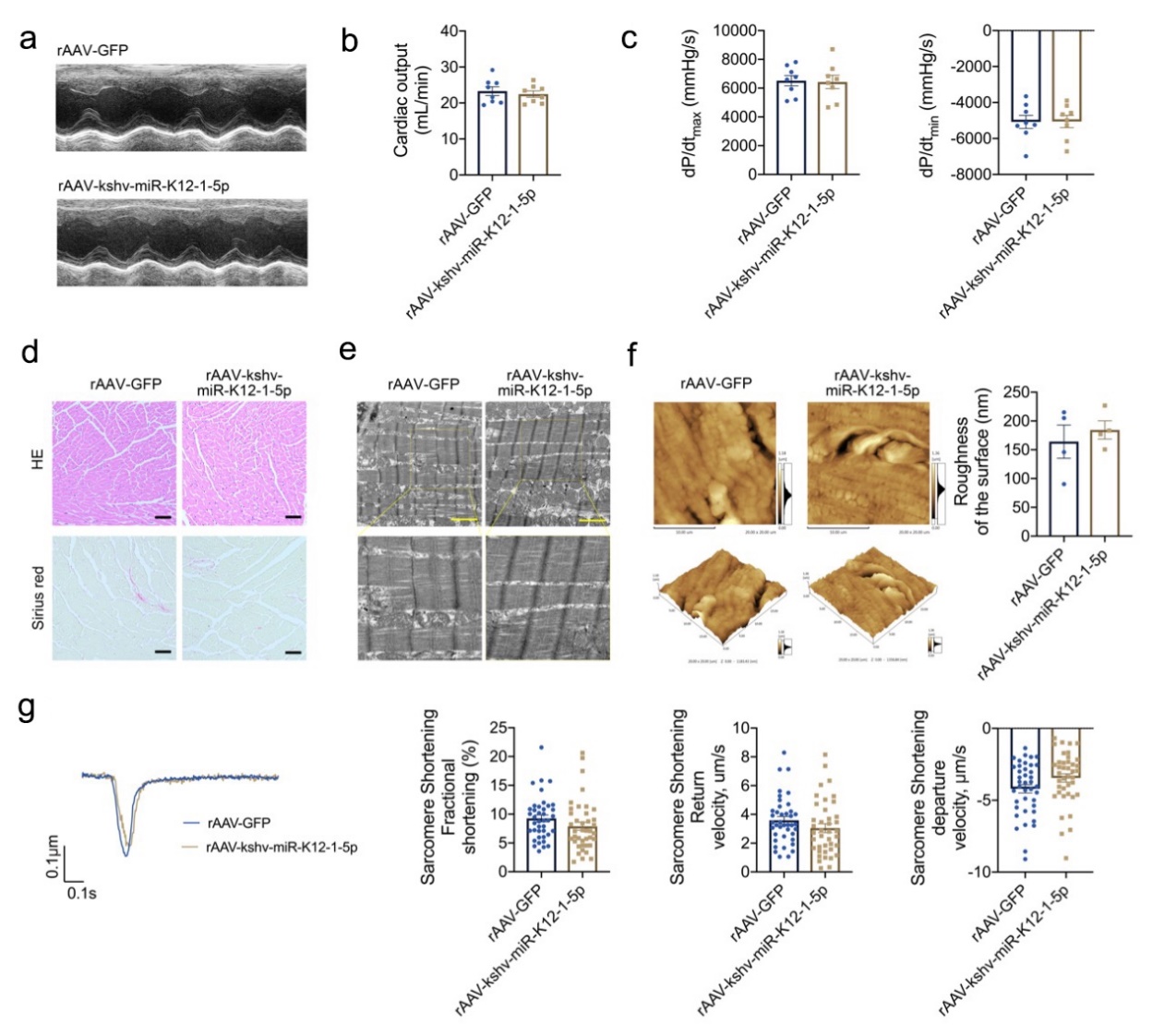


**Supplementary Figure 18**. The mice did not develop DCM after 6-week rAAV-kshv-miR-K12-1-5p treatment. (a) Representative images of M-mode echocardiography in mice. (b) Cardiac output of mice with different treatments measured by echocardiography. (c) Cardiac function in mice measured by in vivo hemodynamics. dP/dt_max_, maximal left ventricular pressure rising rate; dP/dt_mjn_, minimal left ventricular pressure rising rate. (d) HE staining (up) and Sirius red staining (down) of representative heart tissue sections of mice with different treatments. Scale bar=100µm. (e) Transmission electron microscope of mice with different treatments. Scale bar=2µm. (f) 3D atomic force microscopy images (left) and the roughness of the surface (right) of heart samples in mice with different treatment. (g) Representative sample traces, sarcomere shortening fractional shortening, return velocity, and departure velocity in mice with different treatment.

**Supplementary Figure 19.**

**
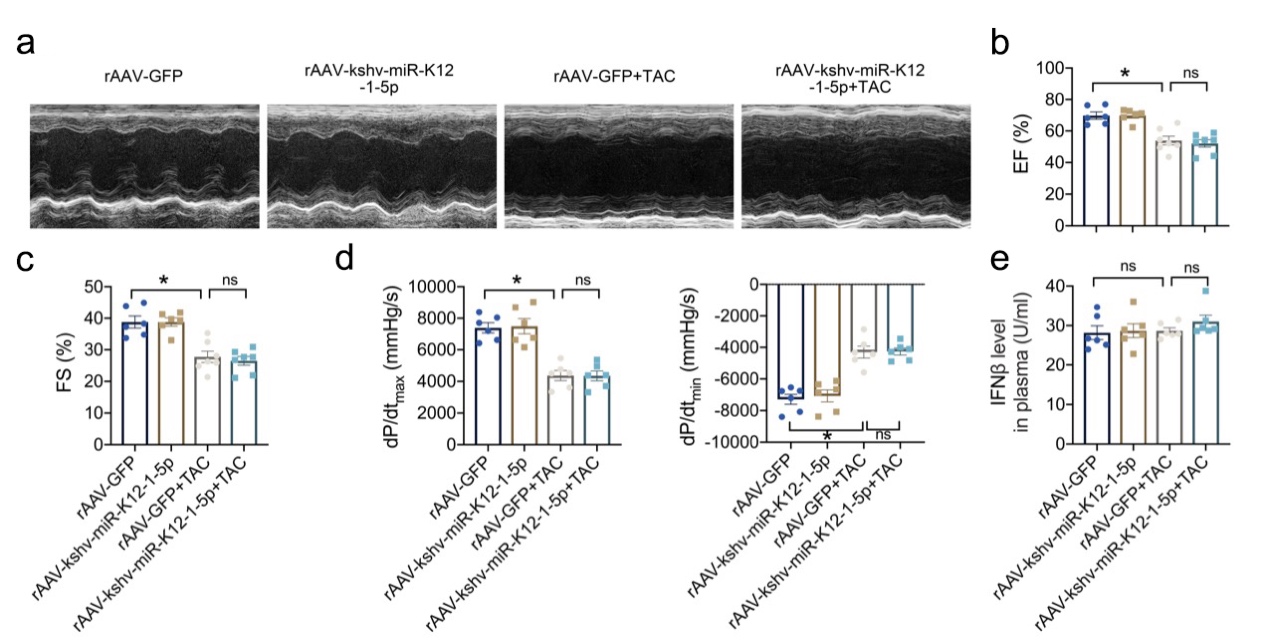
**

**Supplementary Figure 19.** kshv-miR-K12-1-5p showed no effect on cardiac function and IFNβ level in TAC-induced heart failure model. (a) Representative images of M-mode echocardiography in mice underwent rAAV and TAC treatments. (b) Ejection fraction and (c) shortening fraction of mice measured by echocardiography. (d) Cardiac function in mice measured by in vivo hemodynamics. (e) IFNβ levels in plasma detected by ELISA among different groups. *p<0.05.

**Supplementary Figure 20.**


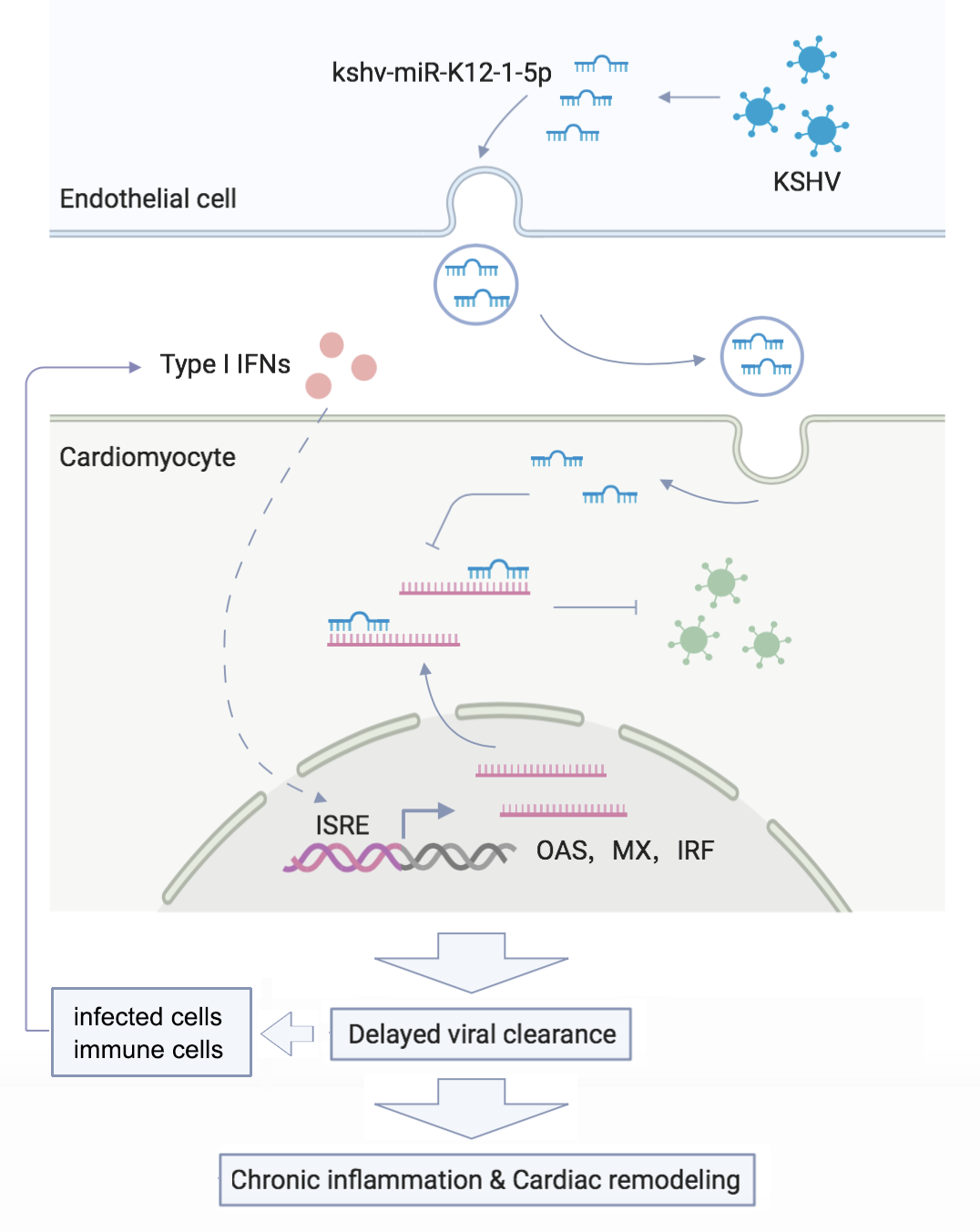


**Supplementary Figure 20.** kshv-miR-K12-1-5p contributed to DCM via blocking type I IFN signaling pathway. Cardiac endothelial cells infected by KSHV could release kshv-miR-K12-1-5p and enter into cardiomyocytes. kshv-miR-K12-1-5p inhibited the expression of anti-viral genes, including OAS, MX and IRF, induced by type I IFNs in cardiomyocytes, which thereby increased subsequent infection with known cardiotropic viruses. The delayed viral clearance contributed to chronic inflammation, cardiac remodeling and DCM development, as well as the production of type I IFNs by the microenvironment feedback mechanisms. ISRE, interferon-sensitive response element. The diagram was generated from BioRender (https://biorender.com).

**Supplementary Tables**

**Supplementary Table 1**. Clinical characteristics of 25 recipients of heart transplantation with DCM and 14 healthy heart sample donors.

| Patient No. | Gender | Age  (years) | Clinical presentation | | | | | | | | | | Primary diagnosis | Other diagnoses |
| --- | --- | --- | --- | --- | --- | --- | --- | --- | --- | --- | --- | --- | --- | --- |
|  |  |  | HR  (b.p.m) | SBP  (mmHg) | DBP (mmHg) | Hb (g/L) | BUN  (mmol/L) | Cr (μmol/L) | CRP  (mg/L) | LVEF  (%) | LVEDD  (mm) | IVS  (mm) |  |  |
| 1 | Male | 54 | 70 | 136 | 83 | 131 | 5.9 | 93.9 | 4.47 | 28 | 71 | 12 | DCM | HPT |
| 2 | Male | 68 | 84 | 93 | 64 | 127 | 12.69 | 103.2 | 7.5 | 31 | 55 | 8 | DCM | - |
| 3 | Male | 42 | 110 | 90 | 65 | 70 | 6.18 | 87.3 | <3.14 | 12 | 70 | - | DCM | - |
| 4 | Male | 47 | 68 | 112 | 76 | 117 | 7.11 | 129.2 | 13.3 | 36 | 73 | 9 | DCM | HPL |
| 5 | Male | 59 | 70 | 95 | 62 | 153 | 5.66 | 73 | <3.14 | 30 | 57 | - | DCM | T2D |
| 6 | Male | 68 | 105 | 95 | 58 | 138 | 18.42 | 171 | 167 | 18 | 81 | 10 | DCM | - |
| 7 | Male | 59 | 82 | 125 | 81 | 138 | 5.99 | 103.2 | 5.86 | 27 | 68 | 10 | DCM | HPT, T2D, HPL |
| 8 | Male | 31 | 80 | 110 | 70 | 126 | 7.32 | 105.3 | <3.14 | 24 | 80 | - | DCM | - |
| 9 | Male | 12 | 86 | 112 | 76 | 113 | 5.47 | 34.7 | 3.45 | 13 | 64 | 6 | DCM | - |
| 10 | Male | 30 | 82 | 109 | 73 | 111 | 16.38 | 124.4 | 12.8 | 14 | 71 | 10 | DCM | - |
| 11 | Male | 49 | 60 | 122 | 78 | 131 | 9.85 | 96.6 | 5.63 | 23 | 55 | 10 | DCM | - |
| 12 | Male | 29 | 72 | 113 | 74 | 142 | 7.76 | 85.1 | 11.3 | 10 | 69 | 10 | DCM | - |
| 13 | Male | 61 | 82 | 113 | 80 | 128 | 8.14 | 88.8 | 3.51 | 26 | 68 | 8 | DCM | T2D |
| 14 | Male | 42 | 58 | 80 | 55 | 135 | 3.94 | 58.6 | 77.2 | 24 | 75 | 9 | DCM | - |
| 15 | Male | 33 | 80 | 110 | 65 | 151 | 6.87 | 107.9 | 10.9 | 18 | 96 | - | DCM | T2D |
| 16 | Male | 62 | 111 | 90 | 71 | 134 | 6.77 | 114.5 | 3.25 | 37 | 62 | 9 | DCM | HPL |
| 17 | Male | 60 | 75 | 108 | 74 | 139 | 9.14 | 24 | 4.58 | 26 | 65 | - | DCM | HPT |
| 18 | Male | 47 | 92 | 83 | 62 | 140 | 9.54 | 92 | <3.14 | 19 | 79 | 7 | DCM | HPL |
| 19 | Male | 58 | 57 | 96 | 58 | 148 | 9.05 | 73.8 | 7.6 | 25 | 70 | 9 | DCM | - |
| 20 | Male | 61 | 80 | 115 | 77 | 138 | 6.74 | 75.8 | 5.43 | 33 | 71 | 7 | DCM | HPL |
| 21 | Male | 58 | 100 | 108 | 86 | 156 | 26.7 | 180.5 | 33 | 16 | 69 | 8 | DCM | HPL |
| 22 | Male | 60 | 78 | 82 | 52 | 113 | 11.95 | 139.6 | 13.9 | 22 | 68 | 9 | DCM | - |
| 23 | Male | 66 | 70 | 106 | 70 | 123 | 7.1 | 98.4 | <3.14 | 22 | 80 | 10 | DCM | - |
| 24 | Male | 59 | 60 | 76 | 52 | 149 | 7.59 | 83.9 | <3.14 | 18 | 72 | 10 | DCM | - |
| 25 | Male | 50 | 84 | 109 | 70 | 85 | 8.06 | 52.8 | 27.1 | 22 | 57 | 8 | DCM | - |
| Donor 1 | Male | 43 | N/A | | | | | | | | | | | |
| Donor 2 | Male | 52 | N/A | | | | | | | | | | | |
| Donor 3 | Male | 39 | N/A | | | | | | | | | | | |
| Donor 4 | Male | 46 | N/A | | | | | | | | | | | |
| Donor 5 | Male | 42 | N/A | | | | | | | | | | | |
| Donor 6 | Male | 36 | N/A | | | | | | | | | | | |
| Donor 7 | Male | 56 | N/A | | | | | | | | | | | |
| Donor 8 | Male | 48 | N/A | | | | | | | | | | | |
| Donor 9 | Male | 33 | N/A | | | | | | | | | | | |
| Donor 10 | Male | 50 | N/A | | | | | | | | | | | |
| Donor 11 | Male | 42 | N/A | | | | | | | | | | | |
| Donor 12 | Male | 37 | N/A | | | | | | | | | | | |
| Donor 13 | Male | 56 | N/A | | | | | | | | | | | |
| Donor 14 | Male | 45 | N/A | | | | | | | | | | | |

DCM, dilated cardiomyopathy; HR, heart rate; b.p.m, beats per minute; SBP, systolic blood pressure; DBP, diastolic blood pressure; Hb, Hemoglobin; BUN, blood urea nitrogen; Cr, creatinine; CRP, C-reactive protein; LVEF, left ventricular ejection fraction; LVEDD, left ventricular end diastolic diameter; IVS, interventricular septum; HPT, Hypertension; T2D, Type 2 diabetes; HPL, Hyperlipidemia.

**Supplementary Table 2.** The list of gene mutations for genetic DCM patients.

| Patient No. | Gene | Transcript | Exon | Coding change | Protein change |
| --- | --- | --- | --- | --- | --- |
| 7 | DSP | NM_004415 | 23 | c.G4071T | p.E1357D |
| 8 | TTN | NM_133379 | 46 | c.T13254G | p.Y4418X |
| 10 | MYBPC3 | NM_000256 | 22 | c.C2265A | p.N755K |
| 15 | MYH7 | NM_000257 | 3 | c.G115A | p.V39M |
| 18 | SGCD | NM_001128209 | 7 | c.C589T | p.R197W |
|  |  | NM_000337 | 8 | c.C592T | p.R198W |
|  |  | NM_172244 | 8 | c.C592T | p.R198W |
| 20 | TTN | NM_003319 | 132 | c.C34726T | p.R11576X |
|  |  | NM_133432 | 133 | c.C35101T | p.R11701X |
|  |  | NM_133437 | 133 | c.C35302T | p.R11768X |
|  |  | NM_133378 | 253 | c.C54217T | p.R18073X |
|  |  | NM_001256850 | 254 | c.C56998T | p.R19000X |
|  |  | NM_001267550 | 304 | c.C61921T | p.R20641X |
|  | LDB3 | NM_001368064 | 11 | c.G1771A | p.E591K |
|  |  | NM_001368065 | 11 | c.G1771A | p.E591K |
|  |  | NM_001080114 | 12 | c.G1630A | p.E544K |
|  |  | NM_001171610 | 12 | c.G1975A | p.E659K |
|  |  | NM_007078 | 12 | c.G1960A | p.E654K |
|  |  | NM_001368066 | 13 | c.G1819A | p.E607K |
| 21 | MYH6 | NM_002471 | 26 | c.C3346T | p.R1116C |
| 22 | TTN | NM_003319 | 118 | c.C29377T | p.R9793X |
|  |  | NM_133432 | 119 | c.C29752T | p.R9918X |
|  |  | NM_133437 | 119 | c.C29953T | p.R9985X |
|  |  | NM_133378 | 239 | c.C48868T | p.R16290X |
|  |  | NM_001256850 | 240 | c.C51649T | p.R17217X |
|  |  | NM_001267550 | 290 | c.C56572T | p.R18858X |
|  | SCN5A | NM_001099405 | 27 | c.G5401A | p.D1801N |
|  |  | NM_001160161 | 27 | c.G5293A | p.D1765N |
|  |  | NM_001354701 | 27 | c.G5398A | p.D1800N |
|  |  | NM_000335 | 28 | c.G5452A | p.D1818N |
|  |  | NM_001099404 | 28 | c.G5455A | p.D1819N |
|  |  | NM_001160160 | 28 | c.G5356A | p.D1786N |
|  |  | NM_198056 | 28 | c.G5455A | p.D1819N |
| 24 | LDB3 | NM_001080115 | 5 | c.C326T | p.P109L |
|  |  | NM_001171610 | 5 | c.C326T | p.P109L |
|  |  | NM_001171611 | 5 | c.C326T | p.P109L |
|  |  | NM_001368063 | 5 | c.C326T | p.P109L |
|  |  | NM_001368064 | 5 | c.C326T | p.P109L |
|  |  | NM_001368065 | 5 | c.C326T | p.P109L |
|  |  | NM_007078 | 5 | c.C326T | p.P109L |
| 25 | LDB3 | NM_001368064 | 10 | c.C1634T | p.P545L |
|  |  | NM_001368065 | 10 | c.C1634T | p.P545L |
|  |  | NM_001080114 | 11 | c.C1493T | p.P498L |
|  |  | NM_001171610 | 11 | c.C1838T | p.P613L |
|  |  | NM_007078 | 11 | c.C1823T | p.P608L |
|  |  | NM_001368066 | 12 | c.C1682T | p.P561L |

**Supplementary Table 3.** Clinical characteristics of patients with KSHV DNA negativity or positivity.

| Variables | non-DCM | | |  | DCM | | |
| --- | --- | --- | --- | --- | --- | --- | --- |
|  | KSHV DNA negativity (n = 852) | KSHV DNA positivity (n = 85) | p value |  | KSHV DNA negativity (n = 543) | KSHV DNA positivity (n = 153) | p value |
| Age, years | 59 ± 11 | 60 ± 13 | 0.171^#^ |  | 56 ± 14 | 58 ± 13 | 0.208^#^ |
| Female, n (%) | 484 (56.8%) | 49 (57.6%) | 0.882* |  | 174 (32.0%) | 73 (47.7%) | <0.001* |
| Smoking, n (%) | 235 (27.6%) | 17 (20.0%) | 0.133* |  | 192 (35.4%) | 53 (34.6%) | 0.869* |
| Heart rate, beats/min | 77 ± 14 | 80 ± 16 | 0.028^#^ |  | 85 ± 19 | 86 ± 17 | 0.414^#^ |
| SBP, mmHg | 133 ± 22 | 135 ± 24 | 0.254^#^ |  | 125 ± 21 | 126 ± 24 | 0.803^#^ |
| DBP, mmHg | 80 ± 13 | 80 ± 14 | 0.934^#^ |  | 79 ± 15 | 81 ± 17 | 0.448^#^ |
| LVEF, % | 60.88 ± 8.83 | 61.36 ± 10.55 | 0.186^#^ |  | 33.83 ± 11.65 | 34.85 ± 12.70 | 0.399^#^ |
| LVEDD, mm | 46.96 ± 4.64 | 47.64 ± 5.97 | 0.492^#^ |  | 65.17 ± 8.33 | 64.64 ± 8.33 | 0.990^#^ |
| NYHA class II, III, or IV | 223 (26.2%) | 37 (45.5%) | <0.001* |  | 506 (93.2%) | 146 (95.4%) | 0.315* |
| Hypertension | 411 (48.2%) | 39 (45.9%) | 0.678* |  | 200 (36.8%) | 60 (39.2%) | 0.590* |
| Type 2 diabetes | 137 (16.1%) | 14 (16.5%) | 0.926* |  | 79 (14.5%) | 25 (16.3%) | 0.583* |
| Hyperlipidemia | 105 (12.3%) | 5 (5.9%) | 0.109* |  | 45 (8.3%) | 16 (10.5%) | 0.402* |
| Baseline medication |  |  |  |  |  |  |  |
| ACEI/ARB | - | - | - |  | 474 (87.3%) | 142 (92.8%) | 0.062* |
| β-Blocker | - | - | - |  | 355 (65.4%) | 91 (59.5%) | 0.183* |
| Diuretics | - | - | - |  | 490 (90.2%) | 143 (93.5%) | 0.265* |
| Spironolactone | - | - | - |  | 473 (87.1%) | 136 (88.9%) | 0.678* |
| Digoxin | - | - | - |  | 293 (54.0%) | 86 (56.2%) | 0.647* |

DCM, dilated cardiomyopathy; HF, heart failure; SBP, systolic blood pressure; DBP, diastolic blood pressure; LVEF, left ventricular ejection fraction; LVEDD, left ventricular end diastolic diameter; ACEI, angiotensin-converting enzyme inhibitor; ARB, angiotensin II receptor blocker. Age, heart rate, SBP, DBP, LVEF and LVEDD are given as mean ± SD, and other values as number of individuals (n) with percentage (n/N) in parentheses. ^#^ By the Mann-Whitney U test. * By the χ^2 test or Fisher’s exact test.

**Supplementary Table 4.** The comparison between KSHV DNA detection and ELISA assay.

| Patient No. | KSHV DNA | anti-KSHV IgG |
| --- | --- | --- |
| 1 | - | - |
| 2 | - | - |
| 3 | - | - |
| 4 | - | - |
| 5 | - | - |
| 6 | - | - |
| 7 | - | - |
| 8 | - | - |
| 9 | - | - |
| 10 | - | - |
| 11 | - | - |
| 12 | - | - |
| 13 | - | - |
| 14 | - | - |
| 15 | - | - |
| 16 | - | - |
| 17 | - | - |
| 18 | - | - |
| 19 | - | - |
| 20 | - | - |
| 21 | - | - |
| 22 | - | - |
| 23 | - | - |
| 24 | - | - |
| 25 | - | - |
| 26 | - | - |
| 27 | - | - |
| 28 | - | - |
| 29 | - | - |
| 30 | - | - |
| 31 | - | - |
| 32 | - | - |
| 33 | - | - |
| 34 | - | - |
| 35 | - | - |
| 36 | - | - |
| 37 | - | - |
| 38 | - | - |
| 39 | - | - |
| 40 | - | - |
| 41 | - | - |
| 42 | - | - |
| 43 | - | - |
| 44 | - | - |
| 45 | + | - |
| 46 | - | - |
| 47 | - | - |
| 48 | - | - |
| 49 | - | - |
| 50 | - | - |
| 51 | - | - |
| 52 | - | - |
| 53 | - | - |
| 54 | + | - |
| 55 | - | - |
| 56 | + | + |
| 57 | - | - |
| 58 | - | - |
| 59 | + | - |
| 60 | - | - |
| 61 | - | - |
| 62 | - | - |
| 63 | + | + |
| 64 | + | + |
| 65 | - | - |
| 66 | - | - |
| 67 | - | - |
| 68 | - | - |
| 69 | - | - |
| 70 | - | + |
| 71 | - | - |
| 72 | - | - |
| 73 | + | + |
| 74 | - | - |
| 75 | - | - |
| 76 | - | +/- |
| 77 | + | + |
| 78 | - | - |
| 79 | - | - |
| 80 | - | - |
| 81 | - | + |
| 82 | + | + |
| 83 | + | + |
| 84 | + | + |
| 85 | - | - |
| 86 | + | + |
| 87 | - | - |
| 88 | + | + |
| 89 | - | - |
| 90 | - | - |

“-”, negative; “+/-”, suspicious; “+”, positive.

**Supplementary Table 5.** Clinical characteristics of patients included in the propensity score-matched analysis.

| Variables | non-DCM (n = 502) | DCM (n = 502) | p value |
| --- | --- | --- | --- |
| Age, years | 58 ± 13 | 59 ± 12 | 0.455^#^ |
| Female, n (%) | 247 (49.2%) | 242 (48.2%) | 0.801* |
| Smoking, n (%) | 103 (20.5%) | 95 (18.9%) | 0.579* |
| Heart rate, beats/min | 77 ± 15 | 84 ± 18 | <0.001^#^ |
| SBP, mmHg | 132 ± 22 | 125 ± 22 | <0.001^#^ |
| DBP, mmHg | 80 ± 13 | 80 ± 15 | 0.822^#^ |
| LVEF, % | 60.67 ± 9.21 | 34.35 ± 12.00 | <0.001^#^ |
| LVEDD, mm | 46.89 ± 4.86 | 64.54 ± 8.16 | <0.001^#^ |
| NYHA class II, III, or IV | 137 (27.3%) | 470 (93.6%) | <0.001* |
| Hypertension | 212 (42.2%) | 222 (44.2%) | 0.566* |
| Type 2 diabetes | 69 (13.7%) | 81 (16.1%) | 0.330* |
| Hyperlipidemia | 65 (12.9%) | 52 (10.4%) | 0.238* |

DCM, dilated cardiomyopathy; HF, heart failure; SBP, systolic blood pressure; DBP, diastolic blood pressure; LVEF, left ventricular ejection fraction; LVEDD, left ventricular end diastolic diameter. Age, heart rate, SBP, DBP, LVEF and LVEDD are given as mean ± SD, and other values as number of individuals (n) with percentage (n/N) in parentheses. ^#^ By the Mann-Whitney U test. * By the χ^2 test or Fisher’s exact test.

**Supplementary Table 6.** Clinical characteristics of patients across the kshv-miR-K12-1-5p level categorical.

| Variables | Q1 | Q2 | Q3 | Q4 | p value |
| --- | --- | --- | --- | --- | --- |
|  | (n=408) | (n=409) | (n=408) | (n=408) |  |
| Age, years | 58±12 | 58±12 | 57±13 | 58±13 | 0.564^#^ |
| Female, n (%) | 205 (50.2%) | 182 (44.5%) | 188 (46.1%) | 205 (50.2%) | 0.239* |
| Smoking, n (%) | 110 (27.0%) | 129 (31.5%) | 127 (31.1%) | 131 (32.1%) | 0.363* |
| Heart rate, beats/min | 80±16 | 80±16 | 81±18 | 82±17 | 0.178^#^ |
| SBP, mmHg | 130±21 | 132±22 | 128±22 | 129±23 | 0.018^#^ |
| DBP, mmHg | 80±14 | 80±14 | 80±15 | 80±15 | 0.614^#^ |
| LVEF, % | 53.40±16.05 | 49.61±16.32 | 46.43±16.74 | 48.72±17.42 | <0.001^#^ |
| LVEDD, mm | 51.91±9.99 | 54.32±10.80 | 57.06±11.39 | 55.53±11.38 | <0.001^#^ |
| NYHA class II, III, or IV | 158 (38.7%) | 226 (55.3%) | 275 (67.4%) | 253 (62.0%) | <0.001* |
| Hypertension | 172 (42.2%) | 164 (40.1%) | 179 (43.9%) | 195 (47.8%) | 0.150* |
| Type 2 diabetes | 61 (15.0%) | 69 (16.9%) | 62 (15.2%) | 63 (15.4%) | 0.877* |
| Hyperlipidemia | 37 (9.1%) | 42 (10.3%) | 45 (11.0%) | 47 (11.5%) | 0.688* |

Q1, the first (lowest) quartile; Q2, the second quartile; Q3, the third quartile; Q4, the fourth (highest) quartile; SBP, systolic blood pressure; DBP, diastolic blood pressure; LVEF, left ventricular ejection fraction; LVEDD, left ventricular end diastolic diameter. Log-transformed expression of kshv-miR-K12-1-5p, age, heart rate, SBP, DBP, LVEF and LVEDD are given as mean ± SD, and other values as number of individuals (n) with percentage (n/N) in parentheses. ^#^ By the Kruskal-Wallis test. * By the linear by linear association $\chi^{2}$ statistic.

**Supplementary Table 7.** The associations between DCM and kshv-miR-K12-1-5p levels in plasma.

| kshv-miR-K12-1-5p level categorical | OR (95% CI) | p value | Adjusted OR (95% CI) | Adjusted p value | p for trend |
| --- | --- | --- | --- | --- | --- |
| Q1 | Reference | - | Reference | - | <0.001 |
| Q2 | 1.74 (1.30-2.33) | <0.001 | 1.17 (0.14-9.67) | 0.885 |  |
| Q3 | 2.88 (2.16-3.85) | <0.001 | 1.57 (1.22-2.01) | <0.001 |  |
| Q4 | 2.19 (1.64-2.92) | <0.001 | 1.36 (1.14-1.62) | 0.001 |  |

DCM, dilated cardiomyopathy; OR, odds ratio; CI, confidence interval; Q1, the first (lowest) quartile; Q2, the second quartile; Q3, the third quartile; Q4, the fourth (highest) quartile. The OR and 95% CI were estimated using logistic regression model after adjustment for age, gender, ejection fraction, smoking, drinking, hypertension, diabetes and hyperlipidemia. The p value (unadjusted) for trend across quartiles was estimated using chi-squared test.

**Supplementary Table 8.** Multivariable analysis of the association between kshv-miR-K12-1-5p level and outcomes of DCM patients.

| Outcomes | kshv-miR-K12-1-5p level categorical | Number of patients with event (%) | Adjusted HR (95% CI) | Adjusted  p value | p for trend |
| --- | --- | --- | --- | --- | --- |
| Death from cardiovascular causes or heart transplantation | Q1 | 22 (21.4) | Reference | - | 0.007 |
|  | Q2 | 50 (32.2) | 1.76 (1.06-2.91) | 0.028 |  |
|  | Q3 | 64 (31.8) | 1.71 (1.05-2.79) | 0.032 |  |
|  | Q4 | 66 (40.0) | 2.00 (1.24-3.25) | 0.005 |  |

DCM, dilated cardiomyopathy; HR, hazard ratio; CI, confidence interval; Q1, the first (lowest) quartile; Q2, the second quartile; Q3, the third quartile; Q4, the fourth (highest) quartile. The hazard ratios and 95% confidence intervals were estimated using Cox Proportional-Hazards Regression model after adjustment for age, gender, ejection fraction, smoking, drinking, hypertension, diabetes and hyperlipidemia. The p value (unadjusted) for trend of hazard ratios across quartiles was calculated using Cox Proportional-Hazards Regression model after merging Q2 and Q3 into one group to satisfy the proportional hazard assumption.

**Supplementary Table 9.** Detection of viral genomes in DCM heart samples.

|  | DCM | |
| --- | --- | --- |
|  | KSHV DNA negativity | KSHV DNA positivity |
|  | (n = 11) | (n = 14) |
| CVB3 | 1 (9.1%) | 3 (21.4%) |
| PB19 | 0 | 2 (14.3%) |
| HHV6 | 2 (18.2%) | 5 (35.7%) |
| CVB3+HHV6 | 0 | 1 (7.1%) |
| PB19+HHV6 | 0 | 1 (7.1%) |
| CMV | 0 | 0 |
| EBV | 0 | 0 |
| HSV | 0 | 0 |
| HHV7 | 0 | 0 |
| ADV | 0 | 0 |
| H1N1 | 0 | 0 |

CVB3, Coxsackievirus B3; PB19, Parvovirus B19; HHV6, Human herpesvirus 6; CMV, Cytomegalovirus; EBV, Epstein–Barr virus; HSV, Herpes simplex virus; HHV7, Human herpesvirus 7; ADV, Adenovirus; H1N1, influenza A virus subtype H1N1.

**Supplementary Table 10.** Echocardiographic characteristics of mice that underwent agomiR and CVB3 treatments at day 14 post infection.

|  | control Agomir | kshv-miR-K12-1-5p  Agomir | control Agomir  +CVB3 | kshv-miR-K12-1-5p  Agomir+CVB3 | p value |
| --- | --- | --- | --- | --- | --- |
|  | (n=6) | (n=6) | (n=6) | (n=6) |  |
| HR (beats/min) | 541±16 | 538±17 | 478±24 | 466±15 | 0.013* |
| LVPW,d (mm) | 0.73±0.03 | 0.68±0.04 | 0.61±0.02 | 0.65±0.05 | 0.149* |
| LVPW,s (mm) | 1.08±0.04 | 1.19±0.05 | 0.96±0.04 | 1.01±0.07 | 0.036* |
| LVAW,d (mm) | 0.82±0.07 | 0.84±0.05 | 0.77±0.05 | 0.75±0.06 | 0.687* |
| LVAW,s (mm) | 1.44±0.12 | 1.44±0.05 | 1.28±0.06 | 1.21±0.06 | 0.120* |
| LVID,d (mm) | 3.57±0.12 | 3.35±0.09 | 3.14±0.05 | 2.81±0.09 | <0.001* |
| LVID,s (mm) | 2.03±0.16 | 1.65±0.06 | 1.74±0.03 | 1.46±0.12 | 0.010* |

HR, heart rate; LVPW, d, LV posterior wall thickness at diastole; LVPW, s, LV posterior wall thickness at systole; LVAW, d, LV anterior wall thickness at diastole; LVAW, s, LV anterior wall thickness at systole; LVID, d, LV internal diameter at diastole; LVID, s, LV internal diameter at systole. Values are given as mean ± SEM; * By the one-way ANOVA test.

**Supplementary Table 11.** Echocardiographic characteristics of mice that underwent rAAV and CVB3 treatments at day 14 post infection.

|  | rAAV-GFP | rAAV-kshv-miR-K12-1-5p | rAAV-GFP+CVB3 | rAAV-kshv-miR-K12-1-5p+CVB3 | p value |
| --- | --- | --- | --- | --- | --- |
|  | (n=6) | (n=6) | (n=6) | (n=6) |  |
| HR (beats/min) | 561±16 | 556±16 | 473±27 | 521±22 | 0.025* |
| LVPW,d (mm) | 0.76±0.04 | 0.79±0.04 | 0.63±0.09 | 0.61±0.03 | 0.008* |
| LVPW,s (mm) | 1.27±0.07 | 1.23±0.05 | 1.02±0.03 | 0.95±0.07 | 0.001* |
| LVAW,d (mm) | 0.85±0.04 | 0.89±0.08 | 0.81±0.08 | 0.75±0.06 | 0.586* |
| LVAW,s (mm) | 1.46±0.04 | 1.47±0.07 | 1.28±0.06 | 1.22±0.12 | 0.043^#^ |
| LVID,d (mm) | 3.37±0.10 | 3.41±0.09 | 3.16±0.11 | 2.71±0.11 | 0.006^#^ |
| LVID,s (mm) | 1.69±0.11 | 1.87±0.11 | 1.64±0.10 | 1.38±0.16 | 0.085^#^ |

HR, heart rate; LVPW, d, LV posterior wall thickness at diastole; LVPW, s, LV posterior wall thickness at systole; LVAW, d, LV anterior wall thickness at diastole; LVAW, s, LV anterior wall thickness at systole; LVID, d, LV internal diameter at diastole; LVID, s, LV internal diameter at systole; rAAV, recombinant adeno-associated virus. Values are given as mean ± SEM; * By the one-way ANOVA test; ^#^ By the Kruskal-Wallis test.

**Supplementary Table 12.** Echocardiographic characteristics of mice after 6-week rAAV treatment.

|  | rAAV-GFP | rAAV-kshv-miR-K12-1-5p | p value |
| --- | --- | --- | --- |
|  | (n=8) | (n=8) |  |
| HR (beats/min) | 520±22 | 527±17 | 0.820* |
| LVPW, d (mm) | 0.61±0.03 | 0.60±0.02 | 0.800* |
| LVPW, s (mm) | 0.94±0.06 | 0.95±0.03 | 0.884* |
| LVAW, d (mm) | 0.93±0.06 | 0.81±0.04 | 0.103* |
| LVAW, s (mm) | 1.37±0.07 | 1.28±0.07 | 0.381* |
| LVID, d (mm) | 3.99±0.13 | 3.81±0.11 | 0.285* |
| LVID, s (mm) | 2.54±0.22 | 2.36±0.11 | 0.485* |

HR, heart rate; LVPW, d, LV posterior wall thickness at diastole; LVPW, s, LV posterior wall thickness at systole; LVAW, d, LV anterior wall thickness at diastole; LVAW, s, LV anterior wall thickness at systole; LVID, d, LV internal diameter at diastole; LVID, s, LV internal diameter at systole; rAAV, recombinant adeno-associated virus. Values are given as mean ± SEM; * By the one-way ANOVA test.

**Supplementary Table 13.** Echocardiographic characteristics of mice that underwent rAAV and TAC treatments.

|  | rAAV-GFP | rAAV-kshv-miR-K12-1-5p | rAAV-GFP+TAC | rAAV-kshv-miR-K12-1-5p+TAC | p |
| --- | --- | --- | --- | --- | --- |
|  | (n=6) | (n=6) | (n=7) | (n=7) |  |
| HR (beats/min) | 556±28 | 561±13 | 511±32 | 528±14 | 0.402* |
| Velocity (mm/s) | 1207±93 | 1159±69 | 3302±136 | 3493±146 | <0.001^#^ |
| LVPW,d (mm) | 0.80±0.06 | 0.78±0.03 | 0.87±0.07 | 1.05±0.04 | 0.007* |
| LVPW,s (mm) | 1.35±0.07 | 1.35±0.08 | 1.29±0.06 | 1.47±0.07 | 0.304* |
| LVAW,d (mm) | 0.77±0.04 | 0.85±0.05 | 0.95±0.07 | 1.12±0.06 | 0.002* |
| LVAW,s (mm) | 1.37±0.04 | 1.51±0.03 | 1.47±0.10 | 1.64±0.07 | 0.073* |
| LVID,d (mm) | 3.61±0.17 | 3.67±0.12 | 4.17±0.09 | 4.19±0.23 | 0.023^#^ |
| LVID,s (mm) | 2.21±0.13 | 2.24±0.08 | 3.02±0.13 | 3.09±0.22 | <0.001* |

HR, heart rate; Velocity, Velocity in aortic arches; LVPW, d, LV posterior wall thickness at diastole; LVPW, s, LV posterior wall thickness at systole; LVAW, d, LV anterior wall thickness at diastole; LVAW, s, LV anterior wall thickness at systole; LVID, d, LV internal diameter at diastole; LVID, s, LV internal diameter at systole; rAAV, recombinant adeno-associated virus; TAC, transverse aortic constriction. Values are given as mean ± SEM; * By the one-way ANOVA test; ^#^ By the Kruskal-Wallis test.

**Supplementary Table 14.** Sequences of primers used in the detection of viral genomes.

| Primer name | Primer sequence (5’-3’) |
| --- | --- |
| GAPDH-F | GCTCCCTCTTTCTTTGCAGCAAT |
| GAPDH-R | TACCATGAGTCCTTCCACGATAC |
| GAPDH-probe | (6FAM)TCCTGCACCACCAACTGCTTAGCACC(TAMRA) |
| KSHV-F | CCGAGGACGAAATGGAAGTG |
| KSHV-R | GGTGATGTTCTGAGTACATAGCGG |
| KSHV-probe | (6FAM)ACAAATTGCCAGTAGCCCACCAGGAGA(TAMRA) |
| PB19-F | CAAAAGCATGTGGAGTGAGG |
| PB19-R | GTGCTGTCAGTAACCTGTAC |
| HHV6-F | GACCCGAGAGATGATTTTGCG |
| HHV6-R | TCGCGCTTTACTGCGTTTAGG |
| CMV-F | GGACCTATTCGTTTTCACACCTAC |
| CMV-R | GTGACAGACACGGCGTATGG |
| EBV-F | GCAGTTTGTCAGCAGCTACTTC |
| EBV-R | CATGCTCTCGTCCACATCTAAG |
| HSV-F | CCACGAGACCGACATGGAGC |
| HSV-R | GTGCTYGGTGTGCGACCCCTC |
| HHV7-F | CAGAAATGATAGACAGATGTTGG |
| HHV7-R | TAGATTTTTTGAAAAAGATTTAATAAC |
| ADV-F | CAGTGGKCDTACATGCACATC |
| ADV-R | GCGGGCRAAYTGCACSAG |
| H1N1-F | CTCAGCAAATCCTACATTA |
| H1N1-R | TAGTAGATGGATGGTGAAT |

**Supplementary Table 15.** Sequences of primers for gene expression by RT-PCR.

| Gene name | Forward primer (5’-3’) | Reverse primer (5’-3’) |
| --- | --- | --- |
| OAS1 | TGGGAGCGAGGGAGCA | TCTTAAAGCATGGGTAATTCAGC |
| OAS2 | AAGCCCTACGAAGAATGTCAGA | GGCCAGCACCTCGAAAGA |
| OAS3 | TCAAACCCAAGCCACAAGTC | GGCGAAGATGGTCAGCAAT |
| MX1 | CCCAGACGGCATAGCGA | CCTGTAGCCTCCGACCCA |
| IFIT1 | GGTCAAGGATAGTCTGGAGCAA | TCCACTTCAAGCACCTTTTCA |
| IFIT3 | GTTGTGACGGGTAGGACGATAG | GTTGCCCAGGCTGGAGTG |
| IRF7 | CGTCGCTTCGTGATGCTG | GCCTCGCCTGTCGTTAGTG |
| RSAD2 | AGCGTCAACTATCACTTCACTCG | CCAACTTGCCCAGGTATTCTC |
| TLR2 | CACTCAGGAGCAGCAAGCACTG | GCAGGAACAGAGCACAGCACAT |
| IL1R1 | CGACTTCCTCTCCAGCCTTCTCT | AGACAGCCACCACAGCCTCT |
| GAPDH | AGAAGGCTGGGGCTCATTTG | AGGGGCCATCCACAGTCTTC |
| CVB3 | CACACTCCGATCAACAGTCA | GAACGCTTTCTCCTTCAACC |
| EMCV | TCTTGGCCGCTTTGTCTAGA | TGGCTTGGTCTCGACTAGTG |
| Gapdh | AGGCCGGTGCTGAGTATGTC | TGCCTGCTTCACCACCTTCT |

**Supplementary Table 16.** List of antibodies.

| Antibody | Company | Catalog # | Source |
| --- | --- | --- | --- |
| Ago2 | Novus Biologicals (Shanghai, China) | H00027161-M01 | Mouse |
| OAS1 | ABclonal Biotech (Cambridge, MA) | A2530 | Rabbit |
| OAS2 | ABclonal Biotech (Cambridge, MA) | A3316 | Rabbit |
| OAS3 | ABclonal Biotech (Cambridge, MA) | A9481 | Rabbit |
| MX1 | ABclonal Biotech (Cambridge, MA) | A1780 | Rabbit |
| IFIT1 | ABclonal Biotech (Cambridge, MA) | A8551 | Rabbit |
| IFIT3 | ABclonal Biotech (Cambridge, MA) | A3924 | Rabbit |
| IRF7 | ABclonal Biotech (Cambridge, MA) | A0159 | Rabbit |
| RSAD2 | ABclonal Biotech (Cambridge, MA) | A8271 | Rabbit |
| GAPDH | ABclonal Biotech (Cambridge, MA) | AC002 | Mouse |
| CD9 | ABclonal Biotech (Cambridge, MA) | A19027 | Rabbit |
| IFNβ1 | ABclonal Biotech (Cambridge, MA) | A1575 | Rabbit |
| cTNT | ABclonal Biotech (Cambridge, MA) | A10354 | Rabbit |
| KSHV ORF73 | Abcam (Cambridge, MA) | ab4103 | Rat |
| KSHV ORF45 | Abcam (Cambridge, MA) | ab36618 | Mouse |
| cTNT | Abcam (Cambridge, MA) | ab33589 | Mouse |
| CD31 | Cell Signaling Technology (Danvers, MA) | 77699s | Rabbit |
| Col1a1 | Cell Signaling Technology (Danvers, MA) | 84336s | Rabbit |
| Second antibody, HRP | Thermo Fisher scientific (Shanghai, China) | G-21040, G-21234 | Goat |
| Second antibody, Alexa Fluor | Thermo Fisher scientific (Shanghai, China) | A-21206, A-21202, A-21209 | Donkey |
